# Improved therapeutic consistency and efficacy of CD317^+^ MSCs through stabilizing TSG6 by PTX3

**DOI:** 10.1186/s13287-024-03706-3

**Published:** 2024-03-27

**Authors:** Shaoquan Shi, Si Chen, Bowei Liang, Yumeng Li, Qi Ma, Meiqi Li, Jingting Zhang, Lan Yao, Jianyong Xu

**Affiliations:** 1 Shenzhen Key Laboratory of Reproductive Immunology for Peri-Implantation, Shenzhen Zhongshan Institute for Reproduction and Genetics, Shenzhen Zhongshan Obstetrics & Gynecology Hospital, Fuqiang Avenue 1001, Shenzhen, 518060 Guangdong People’s Republic of China; 2 Guangdong Engineering Technology Research Center of Reproductive Immunology for Peri-Implantation, Shenzhen, 518000 People’s Republic of China; 3 Shenzhen University Medical School, Shenzhen University, Shenzhen, 518000 People’s Republic of China

**Keywords:** Mesenchymal stem/stromal cells, MSCs, CD317, Therapeutic consistency, Therapeutic efficacy

## Abstract

**Background:**

Previously, we have demonstrated that the batch variations of human platelet lysate (conventional MSC expansion medium) induce MSC heterogeneity and therapeutic inconsistency. On the other hand, the MSCs expanded with chemical defined medium have improved therapeutic consistency.

**Methods:**

In the current study, we studied the MSC subpopulation composition and variation in different types and batches of MSC expansion medium with scRNA-seq analysis.

**Results:**

MSCs expanded with different batches of media have higher levels of heterogeneity from the perspective of cell subpopulation composition at transcriptome levels and therapeutic inconsistency. The CD317^+^ subpopulation has enhanced immune suppression activities. And the percentage of CD317^+^ MSCs within MSCs is tightly correlated with its immune suppression activities, and also contributes to the heterogeneity and therapeutic inconsistency of MSCs. the CD317^+^ MSCs have increased expression levels of PTX3, which might stabilize the TSG6 protein and improve the therapeutic effects

**Conclusions:**

Thus, purifying CD317^+^ MSCs is one efficient strategy to reduce MSC heterogeneity and increase the therapeutic consistency of MSCs.

**Supplementary Information:**

The online version contains supplementary material available at 10.1186/s13287-024-03706-3.

## Background

Mesenchymal stem/stromal cells (MSCs) are a diverse cell population found in various tissues like bone marrow, umbilical cord, teeth, and adipose tissue. Unlike other adult stem cells or fully differentiated cells, MSCs primarily function to sense and respond to changes in their microenvironment. They adapt to factors like oxygen levels, cytokines, and extracellular matrix composition and respond by modifying the extracellular matrix, recruiting immune cells, and releasing bioactive molecules like cytokines. Their immunomodulatory properties make them valuable in conditions involving immune system dysregulation, such as autoimmune diseases [1]. Additionally, MSCs promote tissue repair and angiogenesis, making them promising for treating diseases like cardiovascular disorders, diabetes, and neurological conditions. Both pre-clinical and clinical studies support their therapeutic potential, positioning MSCs as a key candidate for advancing medical treatments across various diseases with ongoing research expected to further enhance their clinical use [1–5].

After the initial demonstration of MSCs, extensive research spanning decades has been dedicated to exploring their therapeutic applications. Regrettably, despite the fast growth of clinical trials, only a limited number have successfully evolved into practical therapeutic products. The significant challenge of cell heterogeneity stands as a formidable obstacle in the pursuit of anticipated clinical outcomes [1, 2, 4, 6, 7]. This heterogeneity in MSCs can be attributed to a range of factors, including donor conditions such as age, gender, health status, and genetic background, along with tissue origin and the specific strategies employed for MSC isolation and expansion, encompassing variables like digestion enzymes, matrix proteins, cell culture medium, and passage numbers. These factors collectively contribute to the complexity of achieving consistent and predictable results in MSC-based therapies [2, 6–11].

In pursuit of strategies to mitigate MSC heterogeneity and improve therapeutic consistency, the approach of isolating homogeneous subpopulations guided by specific markers has gained prominence for its potential to yield more predictable clinical outcomes [2, 4, 6]. To date, several distinct MSC subpopulations have been developed, each characterized by its unique functions. These functions encompass the regulation of cell adhesion [12–17], facilitation of regeneration [18–23], and modulation of the immune system [11, 24, 25].

In the realm of immune modulation, specific MSC subpopulations have been explored, including TNFAIP6^+^ MSCs and CD200^+^ MSCs. TNFAIP6^+^ MSCs are responsive to inflammatory environments, secreting anti-inflammatory cytokines like TNFAIP6, which leads to immune suppression and reduces MSC death induced by activated immune cells. Surviving MSCs express more anti-inflammatory cytokines, further enhancing their immune suppression capabilities [11, 24]. The therapeutic effects have been validated in the mouse model of acute inflammation [11]. However, the levels of TSG-6 mRNA are negatively correlated with their potential for osteogenic differentiation in vitro and poorly correlated with other criteria for evaluating human MSCs [24]. CD200^+^ subpopulations have demonstrated enhanced immune suppression in a mouse skin allograft model, potentially related to CD200's involvement in dendritic cell and macrophage immune responses, although further validation is required. CD200 is down-regulated during differentiation [26]. And its expression is absent in umbilical cord blood-derived MSCs and minimal in adipose-derived MSCs [26–28]. Therefore, current research on immune regulation by MSC subpopulations remains limited and more MSC specific markers need to be identified.

The emergence and development of high-throughput technologies have revolutionized various fields of life sciences [29–31]. Within the realm of high-throughput technologies, single-cell RNA sequencing (scRNA-seq) has emerged as a significant innovation in the MSC field, garnering widespread interest in recent years. By unraveling the gene expression profiles of individual cells within a population, this technique unveils the remarkable intricacies of cellular diversity and heterogeneity, ushering in a revolutionary breakthrough in cellular biology research [32, 33]. Unlike traditional bulk RNA sequencing methods, scRNA-seq offers precise insights into cell functions and types, all while overcoming the challenges posed by sample heterogeneity [33–35]. Through scRNA-seq, various new subpopulations of MSCs have been unveiled. These include CMKLR1^+^ MSCs with enhanced immune suppression capabilities [36], LRRC75A^+^ MSCs producing increased levels of VEGF [37], and S100A9^+^ and F3^+^ MSCs demonstrating superior regenerative properties [38–40].

Previously, we have demonstrated that the batch variations of human platelet lysate (conventional MSC expansion medium) induce MSC heterogeneity and therapeutic inconsistency [9]. On the other hand, the MSCs expanded with chemical defined medium have improved therapeutic consistency [9]. To further investigate whether the MSC subpopulation variations are involved in the induction of MSC heterogeneity and therapeutic inconsistency by the batch variation of MSC expansion medium, in the current study, we studied the MSC subpopulation composition and variation in different types and batches of MSC expansion medium with scRNA-seq analysis. Our data revealed that the CD317^+^ MSCs have improved immune suppression activities and therapeutic effects in the mouse model of IBD (inflammatory bowel disease). Furthermore, the percentage of CD317^+^ MSCs could be an efficacy predictor of its therapeutic effects in vivo.

## Methods

### Human MSCs isolation, expansion and purification

This study was approved by the ethics committee of Shenzhen Zhongshan Urology Hospital and Shenzhen University, and followed the tenants of the Declaration of Helsinki. The human umbilical cord derived MSCs were isolated, expanded and characterized as described previously [8–11]. Briefly, the MSCs were isolated from human umbilical cords (donor age: 28 years old) after the tissue was minced and digested with 1 mg/mL collagenase B (STEMCELL Technologies). MSCs were expanded with chemical defined medium NBVbe we developed [10] or different batches of PL plus 2 U/ml heparin [9]. The MSCs were characterized with StemPro® Adipogenesis Differentiation Kit (Gibco), StemPro® Osteogenesis Differentiation Kit (Gibco), StemPro® Chondrogenesis Differentiation Kit (Gibco) according to instructions. The CD317^+^ and CD317^−^ MSCs were purified with BD FACSAria SORP cell sorter (BD Biosciences) after staining with anti-CD317-PE (Thermo Fisher Scientific) or IgG-PE. For PTX3 overexpression, the coding region sequence was cloned into the lentiviral vector (pLVX-Puro) as described before [8]. For shRNA construction, target sequences were cloned into the lentivirus pLKO.1-puro vector as described before [41]. Lentivirus production and cell infection were performed as described previously [8]. Target sequences of PTX3 were listed in Additional file 1: Table S1.

### Single-cell RNA-seq and analysis

Human MSCs were prepared for scRNA-seq (single-cell RNA sequencing) at passage 6 at 90% confluence. Cells were detached with TrypLE and resuspended in HBSS containing 0.04% BSA at the concentration of 1 × 10^6^ cells/mL. Samples (1 × 10^6^ cells for each sample) were sequenced with the Illumina NovaSeq 6000 System (paired-end mode) after library construction with 10 × Genomics Chromium platform. Sequencing data were analyzed using Seurat packages in R (v 4.0.0) after being processed with 10xGenomics pipeline Cell Ranger (v2.1.0).

### CD317^+^/CD317^−^ MSC purification

Human MSCs were prepared for cell purification at passage 3. The MSCs were detached with TrypLE and stained with anti-CD317-PE (Thermo Fisher Scientific) or IgG-PE. Then, the CD317^+^ and CD317^−^ MSCs were purified with the BD FACSAria SORP cell sorter (BD Biosciences).

## MSC-PBMC co-culture

The MSC-PBMC co-culture was performed as described previously with modifications [8, 9, 11]. Briefly, the human PBMCs (peripheral blood mononuclear cells) were isolated with the Ficoll-Paque® Plus (Merk). The PBMCs were stained with the CellTrace™ CFSE (ThermoFisher), stimulated with Dynabeads® Human T-Activator CD3/CD28 (Thermo Fisher Scientific) for 24 h, and co-cultured with purified CD317^+^ or CD317^−^ MSCs (20 × 10^4^ PBMCs vs 5 × 10^4^ MSCs) for 48 h. The fluorescence signal was detected with flow cytometer (BD Accuri™ C6 Plus).

### Mouse model of IBD (full name)

The mice (C57BL/6J, male, 8 weeks old) were purchased from the Guangdong Medical Laboratory Animal Center and maintained in specific pathogen-free conditions. This study has been reported in line with the ARRIVE guidelines 2.0 and approved by the Animal Research Ethics Committee of the School of Medicine, Shenzhen University. The mouse model of IBD (inflammatory bowel disease) was induced by providing 3% dextran sodium sulfate (DSS) in the drinking water with refreshed every two days. After 7 days treatment, the drinking water was refreshed without DSS. The disease activity index (DAI) was estimated as described before [42]. Mice were divided into 3 groups of eight mice each as follows: Group I, mice transplanted with PBS intravenously; Group II, mice transplanted with CD317^+^ MSCs (1 × 10^6^ cells/mouse) intravenously; Group III, mice transplanted with CD317^−^ MSCs (1 × 10^6^ cells/mouse) intravenously. Serum levels of cytokines were measured with ELISA kits as described before [11]. Quantitative PCR (qPCR) was performed as described before after total RNA extraction and reverse transcription [8, 11]. The primer sequences were listed in Additional file 1: Table S1.

### Tissue analysis

Mice were anesthetized with isoflurane by using the anesthesia system (R550, RWD Life Science) and euthanized with overdose CO_2_. The hematoxylin and eosin (HE) analysis of the colon tissue was performed as described previously [8, 11]. Histology score was estimated as described before [43]. Immunohistochemistry staining of CD45 was performed as described before [11].

### Statistics

Data are shown as mean ± SEM (standard error of the mean) and analyzed with SPSS software for Windows (version 26, SPSS Inc). Student *t*-test was applied to the two groups comparison. One-way ANOVA analysis was applied to the multiple group comparison with normal data distribution, parametric test and Tukey Post Hoc tests. *P* < 0.05 indicates statistical significance.

## Results

**Fig. 1:**
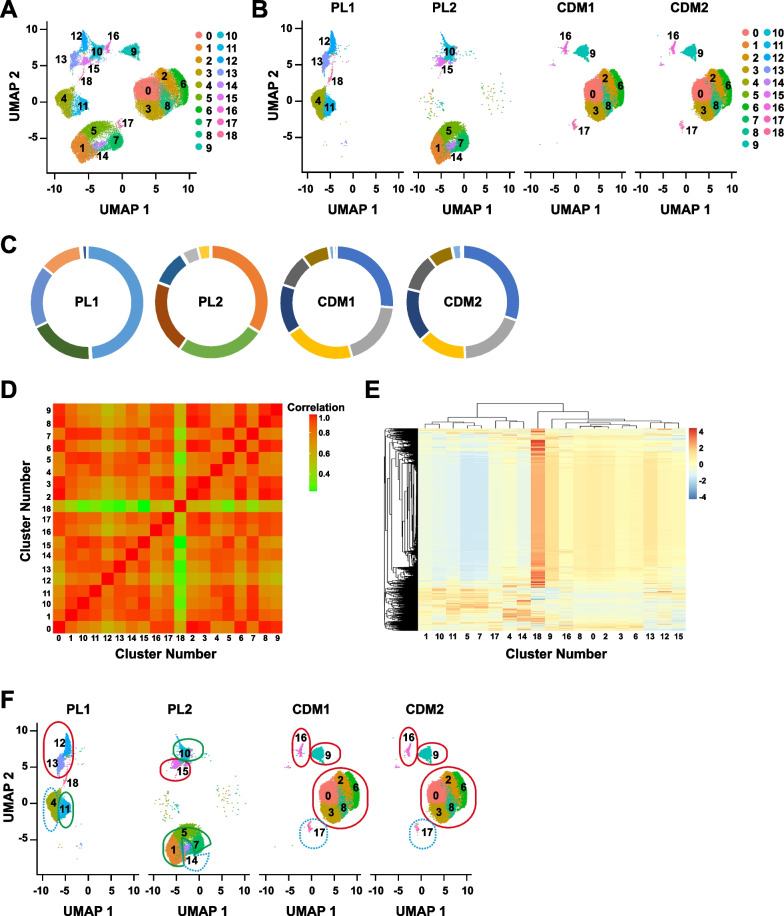
scRNA-Seq of MSCs. The MSCs expanded with two batches of conventional MSC culture medium containing PL (human platelet lysate, PL1 and PL2) and full chemical defined medium (CDM1 and CDM2) were subject to single-cell RNA sequencing (scRNA-seq). **A** Cell cluster identification via non-linear dimensionality reduction analysis with UMAP after integrating 4 samples. **B** Subpopulation distribution and constitution in MSCs expanded with different media. **C** Percentage of different clusters in MSCs expanded with different media. **D** Correlation analysis of different MSC clusters. **E** Clustering analysis of different MSC clusters. **F** The more correlated clusters were labelled with colors

We have demonstrated previously that the MSC expansion medium could induce the heterogeneity and therapeutic inconsistency of MSCs [9]. To further explore the underlying mechanisms, the MSCs expanded with two batches of conventional MSC culture medium containing human platelet lysate (hPL) and two batches of full chemical defined medium were subject to single-cell RNA sequencing (scRNA-seq, Additional file 1: Table S2). There totally 19 different MSC subpopulations were detected with non-linear dimensionality reduction analysis with UMAP (uniform manifold approximation and projection) (Fig. 1A, Additional file 1: Table S3). And their distribution and composition vary significantly among MSCs expanded with different batches of hPL, while they are more stable in the chemical defined medium (Fig. 1B, C). Correlation and heatmap analysis showed that the Subpopulation 0, 2, 3, 6, 8, 9, 12, 13, 15, and 16 were clustered together; Subpopulation 1, 5, 7, 10, and 11 were clustered together; and Subpopulation 4, 14, 17 were clustered together (Fig. 1D–F). Therefore, batch-to-batch variations of hPL-based media could induce MSC heterogeneity from the perspective of subpopulation composition.

**Fig. 2:**
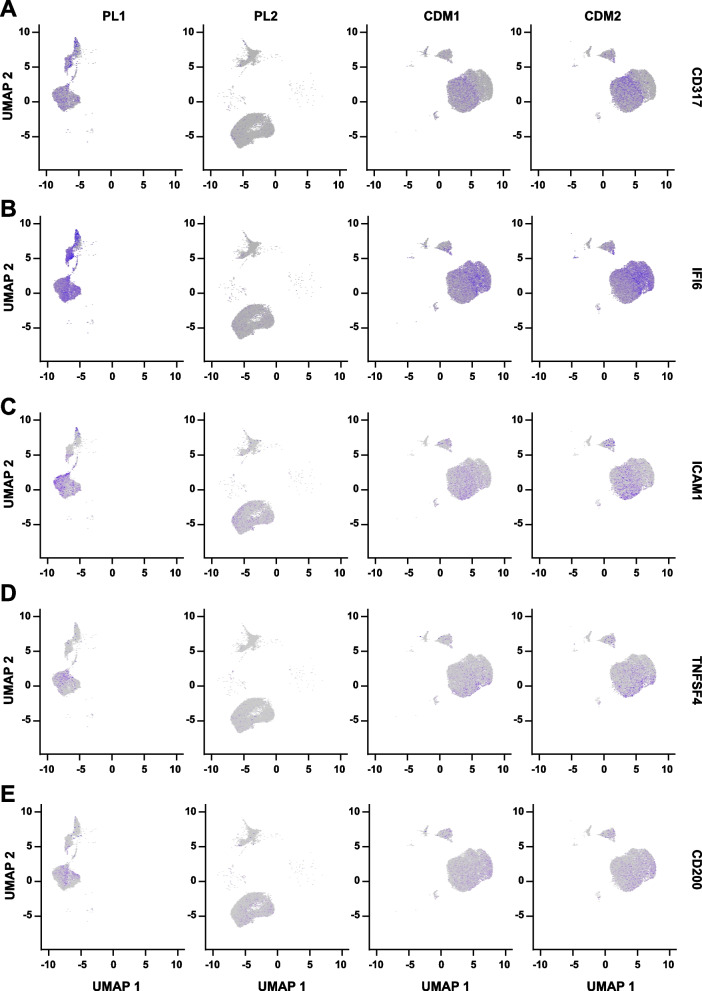
MSC Marker Identification. Feature plotting the gene CD317 (**A**), IFI6 (**B**), ICAM1 (**C**), TNFSF4 (**D**), and CD200 (**E**) on transcriptome of MSCs expanded with different batches of expansion media. IFI6, interferon-α inducible protein 6; ICAM1, intercellular adhesion molecule 1; TNFSF4, TNF Superfamily Member 4

**Fig. 3:**
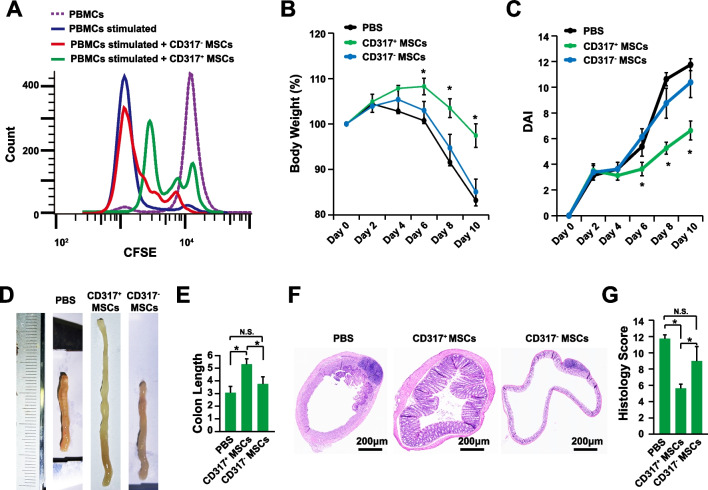
Improved Therapeutic Effects of CD317^+^ MSCs. **A** PBMC proliferation assay after coculture with CD317^+^ or CD317^−^ MSCs. **B** Body weight of IBD mice transplanted with CD317^+^ MSCs, CD317^−^ MSCs, or negative control PBS (n = 8). **C** DAI (disease activity index) of IBD mice transplanted with CD317^+^ MSCs, CD317^−^ MSCs, or negative control PBS (n = 8). **D** Representative figures of colon length. **E** Colon length of IBD mice transplanted with CD317^+^ MSCs, CD317^−^ MSCs, or negative control PBS (n = 8). **F** Representative figures of HE staining of colon tissues after 10 days post DSS stimulation. **G** Histology score of IBD mice transplanted with CD317^+^ MSCs, CD317^−^ MSCs, or negative control PBS (n = 8). *Indicates *P* < 0.05

**Fig. 4:**
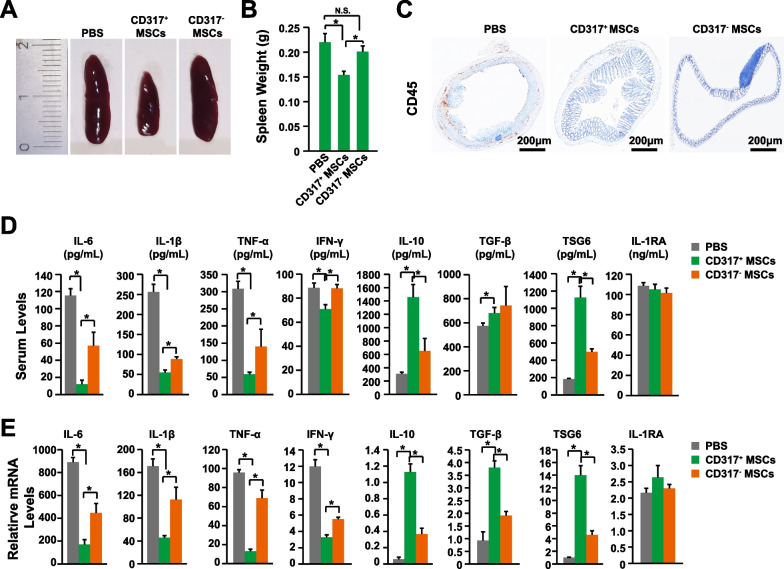
Enhanced Immune-suppression Activities of CD317^+^ MSCs. **A** Representative figures of spleen of IBD mice transplanted with CD317^+^ MSCs, CD317^−^ MSCs, or negative control PBS. **B** Spleen weight of IBD mice transplanted with CD317^+^ MSCs, CD317^−^ MSCs, or negative control PBS (n = 8). **C** Representative figures of CD45 staining of colon tissues after 10 days post DSS stimulation. **D** Serum levels of IL-6, IL-1β, TNF-α, IFN-γ, IL-10, TGF-β, TSG6, and IL-1RA were determined via ELISA (n = 8). **E** mRNA levels of IL-6, IL-1β, TNF-α, IFN-γ, IL-10, TGF-β, TSG6, and IL-1RA in colon tissues were determined via qPCR (n = 8). *Indicates *P* < 0.05

The MSCs expanded with PL1, CDM1, and CDM2 have higher immune suppression activities than the MSCs expanded with PL2 [9]. To identify the MSC subpopulation responsible for immune suppression, a panel of immune related genes were plotted (Additional file 1: Table S4). Among them, the gene CD317, interferon-α inducible protein 6 (IFI6), intercellular adhesion molecule 1 (ICAM1), TNF Superfamily Member 4 (TNFSF4), and CD200 had the similar expression pattern with their immune suppression activities (Fig. 2). The expression of CD317 was restricted to a subset of MSCs with high expression levels (Fig. 2). Furthermore, within the hTERT immortalized human bone marrow MSC colonies, it has been demonstrated that the MSCs from the CD317^+^ colony have up-regulated mRNA levels of immunosuppressive genes than the CD317^−^ MSCs in vitro [44]. And our recent investigation also indicated that the CD317^+^ MSCs have enhanced immunological anti-inflammatory activities [45]. Therefore, the CD317^+^ and CD317^−^ MSCs were purified with fluorescence-activated cell sorting (FACS) from MSCs expanded with hPL. And the MSC-PBMC co-culture assay showed that the CD317^+^ MSCs have stronger immune suppression activities than the CD317^−^ MSCs (Fig. 3A) [45]. To further confirm these findings, the CD317^+^ and CD317^−^ MSCs were transplanted into the mouse model of IBD. The CD317^+^ MSCs showed improved therapeutic effects from the perspectives of body weight (Fig. 3B), DAI (disease activity index, Fig. 3C), colon length (Fig. 3D, E), and histology scoring (Fig. 3F, G). Indeed, the CD317^+^ MSCs had significantly stronger immune suppression activities, from the perspectives of reducing spleen weight (Fig. 4A, B), CD45^+^ lymphocytes infiltration (Fig. 4C), and the expression levels of pro-inflammatory cytokine IL-6, IL-1β, TNF-α, and IFN-γ (Fig. 4D, E). Interestingly, the CD317^+^ MSCs also significantly up-regulated the anti-inflammatory cytokine IL-10, TGF-β, and TSG6 (Fig. 4D, E). Therefore, the CD317^+^ MSCs had stronger immune suppression activities and improved therapeutic effects than CD317^−^ MSCs both in vitro and in vivo.

**Fig. 5:**
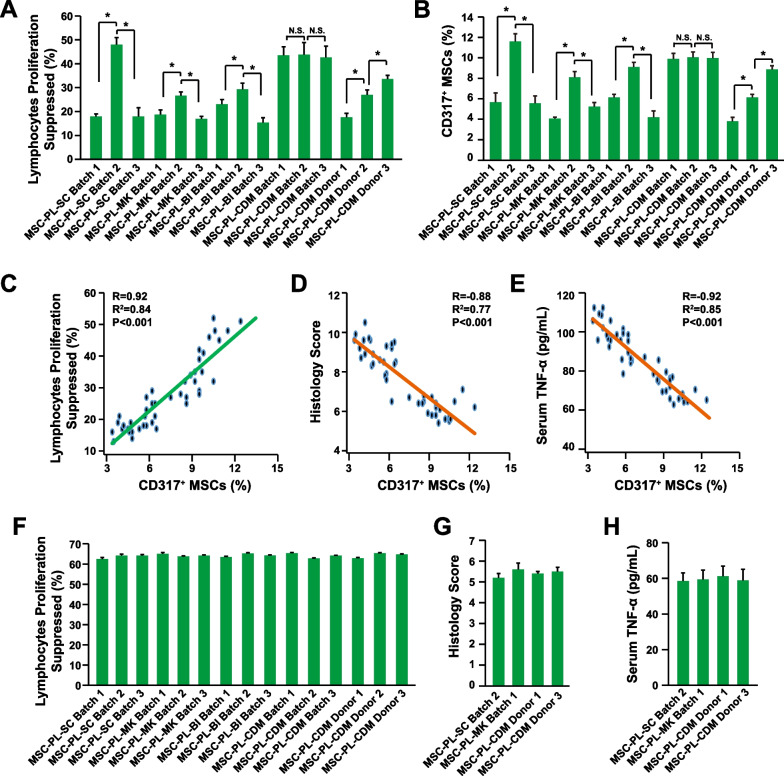
Improved Therapeutic Consistency and Efficacy of CD317^+^ MSCs. The MSCs were expanded with different batches of media or from different donors **A** PBMC proliferation assay after coculture with MSCs (n = 3). **B** Percentage of CD317^+^ MSCs (n = 3). **C** Correlation analysis between lymphocytes proliferation suppressed by MSCs and the percentage of CD317^+^ MSCs within MSCs. **D** Correlation analysis between histology score in IBD mice treated with MSCs and the percentage of CD317^+^ MSCs within MSCs. **E** Correlation analysis between serum levels of TNF-α in IBD mice treated with MSCs and the percentage of CD317^+^ MSCs within MSCs. **F** PBMC proliferation assay after coculture with CD317^+^ MSCs (n = 3). **F** PBMC proliferation assay after coculture with CD317^+^ MSCs (n = 3). **G** Histology score in IBD mice treated with CD317^+^ MSCs (n = 8). **H** Serum levels of TNF-α in IBD mice treated CD317^+^ MSCs (n = 8). *Indicates *P* < 0.05

As we demonstrated before, MSCs expanded with different batches of hPL had different levels of immune suppression activities (Fig. 5A). And the MSCs expanded with chemical defined medium had comparable levels of immune suppression activities (Fig. 5A). Furthermore, the MSCs derived from different donors also had different levels of immune suppression activities (Fig. 5A). Interestingly, the percentage of CD317^+^ MSCs within these MSCs also varied significantly and had the similar pattern with the immune suppression activities (Fig. 5A, B). Indeed, the correlation analysis showed that the percentage of CD317^+^ MSCs within MSCs expanded with different batches of hPL was positively correlated with the ratio of lymphocytes proliferation suppressed (Fig. 5C). In the mouse model of IBD, the percentage of CD317^+^ MSCs was also negatively correlated with the histology score (Fig. 5D) and serum level of TNF-α (Fig. 5E). In contrast, the CD317^+^ MSCs purified from the MSCs expanded with different batches of hPL had the similar level of immune suppression activities in vitro and in vivo (Fig. 5F–H). Therefore, the percentage of CD317^+^ MSCs within MSCs is tightly correlated with its immune suppression activities, and also contributes to the heterogeneity and therapeutic inconsistency of MSCs. Purifying CD317^+^ MSCs is one efficient strategy to reduce MSC heterogeneity and increase the therapeutic consistency of MSCs.

**Fig. 6:**
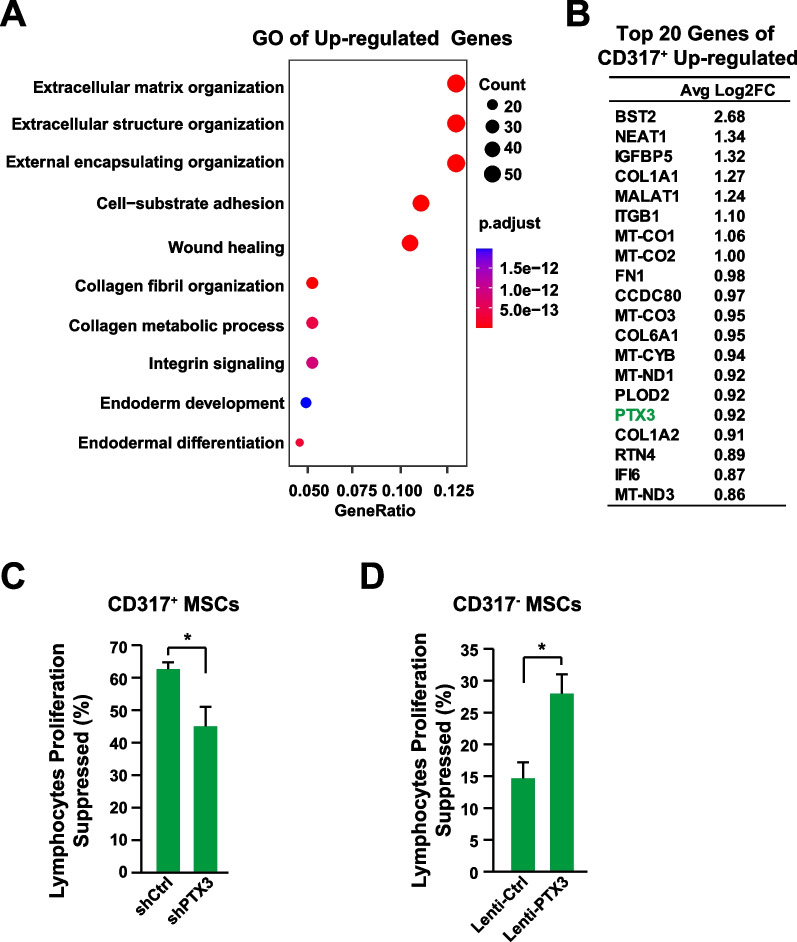
PTX3 Mediates Immune-suppression Function of CD317^+^ MSCs. **A** GO enrichment analysis of up-regulated genes in CD317^+^ MSCs. **B** Top 20 up-regulated genes in CD317^+^ MSCs. **C** PBMC proliferation assay after coculture with CD317^+^ MSCs knocking-down PTX3 (n = 3). **D** PBMC proliferation assay after coculture with CD317^−^ MSCs overexpressing PTX3 (n = 3). GO, gene ontology. *Indicates *P* < 0.05

CD317, also known as BST2 (bone marrow stromal cell antigen 2) or Tetherin, is involved in immunological responses to virus infection and production [46]. The functions and the underlying mechanisms of CD317 in MSCs remain largely unknown. To reveal the underlying mechanisms, the transcriptomes of CD317^+^ MSCs and CD317^−^ MSCs were analyzed (Additional file 1: Table S5). GO analysis showed that the up-regulated genes in the CD317^+^ MSCs were mainly involved in functions such as extracellular matrix modification and wound healing (Fig. 6A). Among the top 20 up-regulated genes in CD317^+^ MSCs (Fig. 6B), the gene PTX3 is potentially interesting. PTX3 (Pentraxin 3), also known as TSG-14 (tumor necrosis factor inducible gene 14), is a soluble pattern recognition molecule which plays critical roles in inflammation and tissue regeneration [47]. Indeed, knocking-down PTX3 in CD317^+^ MSCs significantly reduced the immune suppression activities (Fig. 6C), while overexpressing PTX3 in CD317^−^ MSCs improved their immune suppression activities (Fig. 6D).

**Fig. 7:**
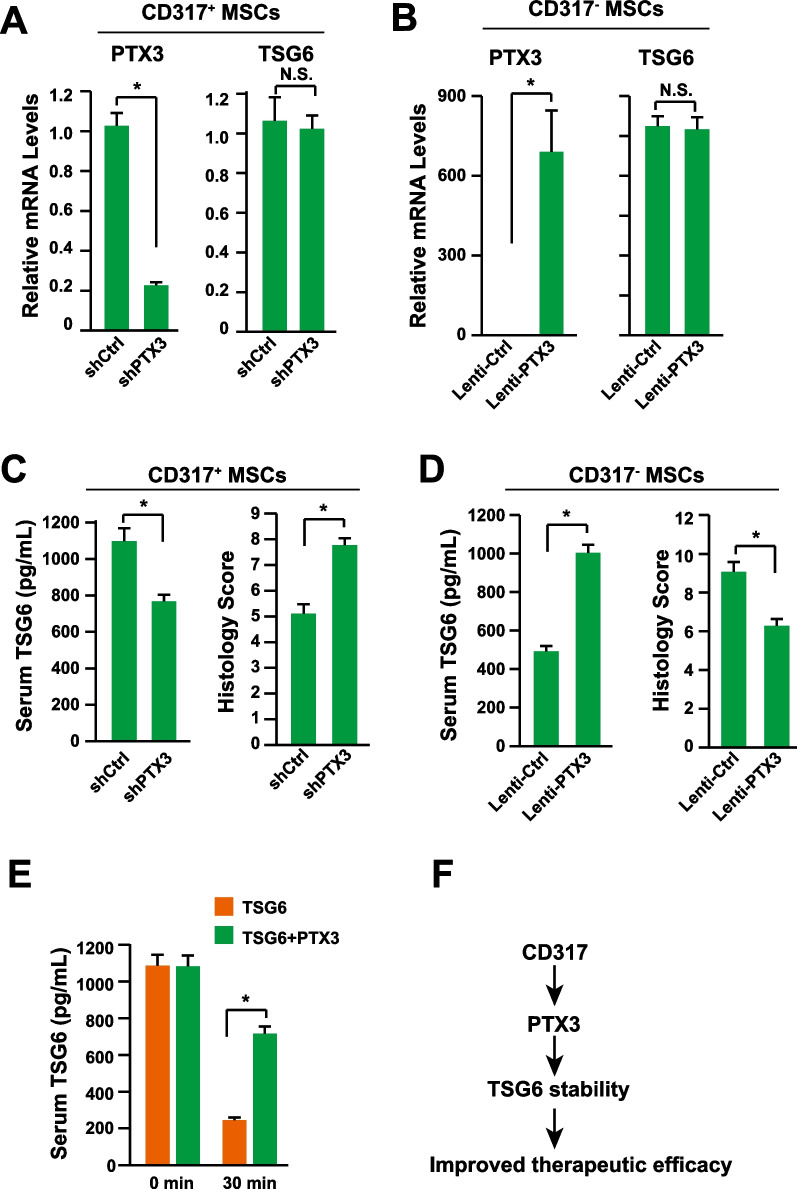
PTX3 Stabilizes TSG6. **A** The mRNA levels of PTX3 and TSG6 were determined via qPCR after knocking-down PTX3 in CD317^+^ MSCs (n = 3). **B** The mRNA levels of PTX3 and TSG6 were determined via qPCR after over-expressing PTX3 in CD317^−^ MSCs (n = 3). **C** Serum level of TSG6 and histology score in IBD mice treated with CD317^+^ MSCs knocking-down PTX3 (n = 8). **D** Serum level of TSG6 and histology score in IBD mice treated with CD317^−^ MSCs over-expressing PTX3 (n = 8). **E** Serum level of TSG6 after intravenously injection of 1 μg TSG6 protein and 1 μg PTX3 protein (n = 8). **F** Proposed potential mechanism of upregulating PTX3 in CD317^+^ MSCs. *Indicates *P* < 0.05

It has been demonstrated that tumor necrosis factor stimulated gene 6 (TSG6) is the major effector contributing to the therapeutic effects of MSCs in treating IBD with strong immune suppression capabilities [1, 11, 48–51]. Furthermore, TSG6 has been demonstrated as a biomarker to predict the therapeutic efficacy of MSCs in vivo [24]. PTX3 could bind to TSG6 directly [52, 53]. Therefore, we wondered that whether PTX3 could regulate the expression or activity of TSG6. Data showed that neither knocking-down PTX3 in CD317^+^ MSCs nor overexpressing PTX3 in CD317^−^ MSCs could regulate the mRNA levels of TSG6 (Fig. 7A, B). However, in the mouse model of IBD, knocking-down PTX3 in CD317^+^ MSCs reduced the serum level of TSG6 and therapeutic effects (Fig. 7C), while overexpressing PTX3 in CD317^−^ MSCs increased the serum level of TSG6 and therapeutic effects (Fig. 7D), indicating that the PTX3 might regulate the protein stability of TSG6. Indeed, co-transfusion of PTX3 and TSG6 significantly delayed the clearance of TSG6 in vivo (Fig. 7E). Therefore, we proposed the underlying mechanism might be that the increased expression level of PTX3 in the CD317^+^ MSCs stabilize the TSG6 protein, which improves the therapeutic effects (Fig. 7F).

In summary, MSCs expanded with different batches of media have higher levels of heterogeneity from the perspective of cell subpopulation composition at transcriptome levels and therapeutic inconsistency. The CD317^+^ subpopulation has enhanced immune suppression activities. And the percentage of CD317^+^ MSCs within MSCs is tightly correlated with its immune suppression activities, and also contributes to the heterogeneity and therapeutic inconsistency of MSCs. Purifying CD317^+^ MSCs is one efficient strategy to reduce MSC heterogeneity and increase the therapeutic consistency of MSCs. The CD317^+^ MSCs have increased expression levels of PTX3, which might stabilize the TSG6 protein and improve the therapeutic effects.

## Discussion

Inflammatory bowel disease (IBD) is attracting more scientists to investigate its underlying mechanisms and therapeutic approaches, due to its critical roles in the development of colorectal cancer (CRC) with increasing prevalence [54, 55]. CRC has been the third most common cancer diagnosis and the third leading cause of cancer-related deaths for both men and women. It’s worth noting that when looking at overall cancer-related deaths, CRC takes the second spot, and it’s the top cause of cancer-related deaths among men under 50 years old [56]. The pathogenesis of IBD involves intricate immune mechanisms [57, 58]. The standard treatment is limited. A significant breakthrough in recent years has been the introduction of TNF-α inhibitors, which have offered substantial, long-term relief and enhancement of the condition for the majority of IBD patients [59]. However, in clinical trials, up to 40% of patients were observed to have a primary non-response to TNF-α inhibitors, and about 23–46% of patients experienced secondary loss of response after one year of treatment [60]. This suggests that IBD still requires new treatment strategies.

IBD is a kind of inflammation related disease [57, 58]. In light of this, the immunological basis of the IBD implies that MSCs-based cell therapies can be a rational therapeutic modality to alleviate IBD pathological signs due to their capacity to moderate inflammatory responses [61–63]. A growing body of proof suggests MSCs as a promising therapeutic tool for IBD treatment mainly due to their immunomodulatory and anti-inflammatory attributes [64–71]. And its clinical efficacy has been confirmed in clinical trials [61–63, 72–75]. Regrettably, few of them have successfully evolved into practical therapeutic products. The significant challenge of cell heterogeneity stands as a formidable obstacle in the pursuit of anticipated clinical outcomes [1–7]. Among different strategies to reduce the MSC heterogeneity and improve the therapeutic efficacy and consistency [2, 6, 8–10], purifying homogenous MSC populations with enhanced biological functions is one promising approach [2, 4, 6].

We have demonstrated previously that the MSC expansion medium could induce the heterogeneity and therapeutic inconsistency of MSCs [9]. Therefore, in the current study, we studied the MSC subpopulation composition and variation in different types and batches of MSC expansion medium with scRNA-seq analysis. The data have shown that the MSCs are very heterogenous from the perspective of subpopulation distribution and constitution. And their distribution and constitution vary significantly among MSCs expanded with different batches of hPL, while they are more stable in the chemical defined medium. To identify the MSC subpopulation responsible for immune suppression, a panel of immune related genes were analyzed. Among them, the CD317^+^ MSCs represent a MSC subpopulation with enhanced immune suppression activities and improved therapeutic effect in the mouse model of IBD.

CD317 is a transmembrane glycoprotein that plays a role in inhibiting virus replication and regulating the immune system. CD317 expression is induced by type I and type II interferons in response to viral infections, and plays a role in regulating NF-κB signaling, which is important for the host's inflammatory response to viruses [46]. It has been demonstrated that the CD317^+^ colony, within the hTERT immortalized human bone marrow MSC colonies, has up-regulated mRNA levels of immunosuppressive genes than the CD317^−^ MSCs in vitro [44]. And the CD317^+^ circulating progenitors had higher regenerative potentials [76, 77]. However, it's worth noting that freshly isolated CD317^−^ MSCs from human bone marrow show better immune suppression abilities than CD317^+^ MSCs [77]. The discrepancy might result from tissue origin (umbilical cord vs bone marrow), expansion medium (platelet vs fetal bovine serum), and cell population (primary vs immortalized and clonal selected).

Correlation analysis showed that the percentage of CD317^+^ MSCs within MSCs expanded with different batches of hPL was positively correlated with their immune suppression activities in vitro, and also their therapeutic effects in vivo. In contrast, the CD317^+^ MSCs purified from the MSCs expanded with different batches of hPL had the similar level of immune suppression activities. Therefore, the percentage of CD317^+^ MSCs within MSCs is tightly correlated with its immune suppression activities, and also contributes to the heterogeneity and therapeutic inconsistency of MSCs. Purifying CD317^+^ MSCs is one efficient strategy to reduce MSC heterogeneity and increase the therapeutic consistency of MSCs.

Transcriptomic analysis and function validation indicate that the gene PTX3 contributes to the immune suppression function of CD317^+^ MSCs. PTX3 is a soluble glycoprotein, responding to inflammatory cues by peripheral blood leukocytes and myeloid DCs, notably induced by IL-1β, TNF-α, and microbial components [47]. Furthermore, the PTX3 is up-regulated in the IBD development, indicating its important role in IBD [47]. PTX3 contributes to the M1-M2 switch of macrophage mediated by MSCs [78]. Knocking-down PTX3 significantly reduced the immune suppression function of MSCs and their therapeutic effect, indicating a potential marker for improving the therapeutic effects of MSCs [78, 79]. Moreover, the PTX3 and TSG6 are coordinated expressed under inflammation stresses [80], and they two interact with each other [52, 53, 81, 82]. And TSG6 is the major effector contributing to the therapeutic effects of MSCs in treating IBD with strong immune suppression capabilities [1, 11, 48–51]. Furthermore, TSG6 has been demonstrated as a biomarker to predict the therapeutic efficacy of MSCs in vivo [24]. Our data also showed here that the increased expression level of PTX3 in the CD317^+^ MSCs stabilize the TSG6 protein, which improves the therapeutic effects.

TSG6 is a relatively small protein exhibiting a wide range of activities. Its diverse functions include regulating immune and stromal cell activities, contributing to extracellular matrix formation and remodeling, and controlling the association of matrix molecules with cell surface receptors and signaling factors, such as chemokines and bone morphogenetic proteins (BMPs). It is upregulated in response to inflammation and is produced by various cell types, including MSCs. Another unique function is its enzymatic activity in catalyzing the covalent modification of non-sulfated GAG hyaluronan (HA) with heavy chains (HCs) from proteoglycans, leading to the formation of HC-HA complexes, which are essential in processes like ovulation, fertilization, and inflammation, where they either confer tissue protection or contribute to pathological processes [48]. The most studied function of TSG6 in MSCs is its immune suppression activities [49, 50, 83–95]. And our previous investigation indicates that the purified TSG6^+^ mouse MSCs have enhanced immune suppression activities and improved therapeutic effects in the mouse model of acute inflammation [11]. TSG6 is involved in the therapeutic effects of MSCs in treating IBD, such as mucosal barrier recovery through activating endogenous stem cells [96–99], and immune modulation [49, 50, 97, 100–102]. Lacking TSG6 makes mice more vulnerable to IBD development, resulting from dysregulated HA deposit [50, 103]. In addition, TSG6 stimulates a macrophage phenotypic shift from M1 to M2, having an important role in alleviating DSS-induced colitis [50, 100].

## Conclusions

In conclusion, MSCs expanded with different batches of media have higher levels of heterogeneity from the perspective of cell subpopulation composition at transcriptome levels and therapeutic inconsistency. The CD317^+^ subpopulation has enhanced immune suppression activities. And the percentage of CD317^+^ MSCs within MSCs is tightly correlated with its immune suppression activities, and also contributes to the heterogeneity and therapeutic inconsistency of MSCs. Purifying CD317^+^ MSCs is one efficient strategy to reduce MSC heterogeneity and increase the therapeutic consistency of MSCs. The CD317^+^ MSCs have increased expression levels of PTX3, which might stabilize the TSG6 protein and improve the therapeutic effects.

### Supplementary Information

**Additional file 1: Table S1.** Primers and shRNAs. **Table S2.** Seq-analysis inflomation. **Table S3.** Cluster markers top20. **Table S4.** Immunogenes list. **Table S5.** DEGs of CD317posi_vs_nega

## Data Availability

The scRNA-seq dataset has been deposited into the China National Center for Bioinformation (https://www.cncb.ac.cn/) with the accession BioProject No. PRJCA024438.
