# Supplementary material for: Improved therapeutic consistency and efficacy of CD317+ MSCs through stabilizing TSG6 by PTX3
Source: Stem Cell Res Ther. 2024 Mar 27;15:92. doi: 10.1186/s13287-024-03706-3 (PMC10976765; doi:10.1186/s13287-024-03706-3)
Supplement: Supplementary file 1 — Additional file 1: Table S1. Primers and shRNAs. Table S2. Seq-analysis inflomation. Table S3. Cluster markers top20. Table S4. Immunogenes list. Table S5. DEGs of CD317posi_vs_nega [file 13287_2024_3706_MOESM1_ESM.pdf]

Supplementary Table 1. Primers and shRNAs

Name	Forwad 5`--3`	Reverse 5`--3`	Applications
IL-6	TACCACTTCACAAGTCGGAGGC	CTGCAAGTGCATCATCGTTGTTC	
IL-1β	TGGACCTTCCAGGATGAGGACA	GTTCATCTCGGAGCCTGTAGTG	
TNF-α	GGTGCCTATGTCTCAGCCTCTT	GCCATAGAACTGATGAGAGGGA[illegible]	
IFN-γ	CAGCAACAGCAAGGCGAAAAAGG	TTTCCGCTTCCTGAGGCTGGAT	
IL-10	CGGGAAGACAATAACTGCACCC	CGGTTAGCAGTATGTTGTCCAGC	qPCR
TGF-β	TGATACGCCTGAGTGGCTGTCT	CACAAGAGCAGTGAGCGCTGAA	
IL1RA	TGTGCCTGTCTTGTGCCAAGTC	GCCTTTCTCAGAGCGGATGAAG	
TSG6	TCACCTACGCAGAAGCTAAGG	TCCAACTCTGCCCTTAGCCA	
PTX3	CGAAATAGACAATGGACTCCATCC	CTCATCTGCGAGTTCTCCAGCA	
beta-actin	CACCATTGGCAATGAGCGG	AGGTCTTTGCGGATGTCCAC	
shCtrl	CCTAAGGTTAAGTCGCCCTCG		shRNA
shPTX3	GAGGAGCTCAGTATGTTTCAT		

SupplementaryTable 2. Seq-analysis inflomation

Table S2.1. Sequencing Statistics

Sample	Number of Reads	Mean Reads per Cell	Valid Barcodes	Q30 Bases in Barcode	Q30 Bases in RNA	Q30 Bases in Sample Index	Q30 Bases in UMI
PL_1	782,475,198	66,992	98.60%	96.00%	81.80%	92.50%	96.60%
PL_2	792,115,780	49,856	98.50%	96.20%	83.20%	93.50%	96.70%
CDM_1	888,476,328	69,629	95.30%	98.30%	93.50%	96.20%	98.40%
CDM_2	853,683,406	70,071	94.80%	97.80%	88.40%	94.40%	97.90%

Table S2.2. Mapping Statistics

Sample	Reads Mapped Confidently to Transcriptome	Reads Mapped Confidently to Exonic Regions	Reads Mapped Confidently to Intronic Regions	Reads Mapped Confidently to Intergenic Regions	Sequencing Saturation
PL_1	70.10%	74.10%	14.90%	3.00%	49.70%
PL_2	73.10%	77.60%	12.90%	2.70%	35.80%
CDM_1	67.00%	71.70%	18.90%	3.60%	31.90%
CDM_2	66.30%	70.80%	18.10%	3.50%	31.40%

Table S2.3. Expression Statistics

Sample	Estimated Number of Cells	Fraction Reads in Cells	Mean Reads per Cell	Median Genes per Cell	Total Genes Detected	Median UMI Counts per Cell
PL_1	11,680	80.80%	66,992	3,332	22,791	12,920
PL_2	15,888	89.50%	49,856	4,847	22,309	21,298
CDM_1	12,760	94.50%	69,629	4,916	23,063	27,549
CDM_2	12,183	92.60%	70,071	4,781	22,811	26,737

Supplementary Table 3 Cluster markers top20

gene	p_val	avg_logFC	pct.1	pct.2	p_val_adj
RBP1	0	0.85978003	0.994	0.595	0
RPS20	0	0.808543794	1	0.91	0
TGFBI	0	0.7838048	1	0.874	0
RPL13A	0	0.737719094	1	0.955	0
MALAT1	0	0.708056032	1	0.958	0
MDK	0	0.694951716	0.995	0.82	0
IGFBP3	0	0.673550716	0.954	0.444	0
FBLN1	0	0.646403315	0.992	0.746	0
CHI3L1	0	0.640806324	0.613	0.292	0
DCN	0	0.635179512	1	0.898	0
CRABP2	0	0.631325275	0.776	0.307	0
RPL27A	0	0.629281553	1	0.968	0
CLU	0	0.626487529	0.984	0.672	0
RPS11	0	0.62183658	1	0.955	0
RPL23	0	0.613047924	1	0.957	0
RPL37A	0	0.590818516	1	0.975	0
POSTN	0	0.583121388	0.73	0.361	0
RPS2	0	0.554997814	1	0.99	0
RPS27	0	0.546343038	1	0.977	0
QPRT	0	0.537718905	0.978	0.677	0
CXCL6	0	0.854151471	0.907	0.563	0
CXCL1	0	0.829946433	0.846	0.487	0
TMEM158	0	0.774955452	0.999	0.844	0
TFPI2	0	0.745256722	0.994	0.841	0
PLAU	0	0.660001619	0.906	0.398	0
ANGPTL4	0	0.654564484	0.951	0.322	0
S100A4	0	0.642483454	0.98	0.88	0
CXCL8	0	0.617925825	0.662	0.28	0
SPON2	0	0.588783623	0.992	0.946	0
HMGA1	0	0.57636532	0.999	0.933	0
PHLDA1	0	0.558318559	1	0.823	0
ITGA2	0	0.545765659	0.989	0.639	0
CTSL	0	0.524079462	0.998	0.898	0
MLLT11	0	0.522157493	0.962	0.651	0
WNT5A	0	0.499368678	0.973	0.573	0
JUNB	0	0.486332198	0.948	0.686	0
ATP5MC3	0	0.485518128	0.999	0.946	0
ARHGAP22	0	0.48505093	0.971	0.555	0
SDF2L1	0	0.484797103	0.976	0.722	0
TUBA1B	0	0.483488515	0.999	0.947	0
CHI3L11	0	1.196873186	0.768	0.292	0
LUM1	0	1.127501378	1	0.903	0
DCN1	0	0.938554223	1	0.903	0
IGFBP31	0	0.813325011	0.976	0.468	0
CLU1	0	0.809528959	0.995	0.687	0
ALDH1A1	0	0.686892716	0.717	0.253	0
RPS201	0	0.660950975	1	0.915	0
RPL13A1	0	0.646144093	1	0.957	0
COL1A11	0	0.626005143	1	0.957	0
FGF71	0	0.625665276	0.987	0.738	0
COL3A11	0	0.604291801	1	0.947	0
TGFBI1	0	0.582142727	1	0.88	0

C1R1	0	0.572966227	0.986	0.537	0
SERPINF11	0	0.569023642	0.973	0.525	0
IGFBP51	0	0.56207143	0.976	0.752	0
NUPR11	0	0.558083179	1	0.799	0
MALAT11	0	0.55649804	1	0.96	0
IGFBP61	0	0.550226121	0.999	0.83	0
SERPINE2	0	0.543158129	0.999	0.916	0
C1S1	0	0.540512699	0.98	0.575	0
PTX31	0	1.076565612	0.997	0.864	0
KRT181	0	0.801173071	0.967	0.777	0
TPM1	0	0.797972282	1	0.939	0
IGFBP52	0	0.782038517	0.956	0.757	0
RHOBTB31	0	0.775617613	0.999	0.871	0
CALD11	0	0.736275043	1	0.952	0
ID31	0	0.695062709	0.91	0.552	0
TAGLN	0	0.649246685	1	0.957	0
KRT191	0	0.616104477	0.883	0.558	0
CTGF	0	0.586240011	0.96	0.656	0
RPS202	0	0.572796782	1	0.916	0
CSRP21	0	0.56536338	0.986	0.712	0
RPL13A2	0	0.557151416	1	0.957	0
DAB2	0	0.554854232	0.991	0.743	0
ACTA2	0	0.554311116	0.961	0.693	0
ANXA1	0	0.541451162	1	0.945	0
TLN1	0	0.523324964	0.999	0.832	0
ARID5B1	0	0.522100284	0.988	0.759	0
MYL9	0	0.513849095	0.999	0.932	0
ALDH1A11	7.93E-196	0.509842343	0.476	0.28	1.75E-191
GREM1	0	1.599955987	0.948	0.412	0
RGS4	0	1.556245586	0.922	0.205	0
PLOD2	0	1.432582254	0.996	0.918	0
SERPINB2	0	1.431968679	0.766	0.084	0
DCBLD21	0	1.363788363	0.997	0.873	0
RTN4	0	1.290846916	0.999	0.966	0
PAPPA	0	1.278757795	0.931	0.571	0
SLC20A11	0	1.273877841	0.992	0.776	0
HMGA21	0	1.241696577	0.994	0.704	0
FRMD6	0	1.219456865	0.986	0.758	0
SERPINE1	0	1.214723938	0.998	0.889	0
THBS1	0	1.210347746	0.974	0.766	0
F3	0	1.135719336	0.953	0.483	0
C12orf75	0	1.123703044	0.996	0.948	0
TGFBR2	0	1.109430548	0.976	0.633	0
ATP2B1	0	1.095965956	0.991	0.87	0
PRNP	0	1.073187812	0.984	0.735	0
DNAJB4	0	1.061143247	0.971	0.572	0
MT2A1	0	1.046852572	1	0.997	0
SMURF2	0	1.036706086	0.974	0.626	0
PTTG1	0	1.227324773	0.998	0.579	0
TUBB4B	0	1.217113316	1	0.837	0
H2AFZ	0	1.165960116	1	0.961	0
UBE2S	0	1.163161803	0.999	0.83	0
PLAU1	0	1.151474367	0.976	0.407	0
CCNB1	0	1.140585478	0.975	0.25	0
CDC20	0	1.075471298	0.961	0.167	0
STMN1	0	1.047429721	0.999	0.834	0

CKS2	0	1.044082994	0.962	0.556	0
TUBA1B1	0	0.988279138	1	0.948	0
BIRC5	0	0.896986149	0.98	0.256	0
CKS1B	0	0.889141241	0.987	0.557	0
HMGN2	0	0.872183678	1	0.925	0
RANBP11	0	0.841531794	1	0.905	0
PCLAF	0	0.832832535	0.999	0.565	0
HMGB1	0	0.806396156	1	0.945	0
RAN	0	0.803613627	1	0.961	0
MAD2L1	0	0.792144354	0.938	0.274	0
DTYMK	0	0.788947308	0.995	0.624	0
TUBA1C	0	0.784095509	1	0.897	0
CCDC803	0	1.323996539	1	0.82	0
ACTA21	0	1.315303332	0.966	0.696	0
SERPINE21	0	1.26576597	1	0.918	0
SCRG11	0	0.983956143	0.885	0.24	0
LUM2	0	0.973296875	1	0.905	0
VCAN1	0	0.96052029	1	0.912	0
EFEMP1	0	0.958312087	0.941	0.329	0
TPM11	0	0.901709796	1	0.94	0
TAGLN1	0	0.861885213	1	0.958	0
TGM21	0	0.790610304	0.971	0.705	0
MGP	0	0.78988277	0.672	0.145	0
DCN2	0	0.742152022	1	0.905	0
TGFBI2	0	0.708672841	1	0.883	0
COL1A13	0	0.686048938	1	0.958	0
HLA-B1	0	0.671810543	0.999	0.818	0
LXN2	0	0.643744687	0.968	0.552	0
KRT82	0	0.642780979	0.991	0.728	0
RPL13A3	0	0.639459696	1	0.958	0
SERPINF12	0	0.635068477	0.979	0.535	0
GPC61	0	0.630773324	0.964	0.425	0
UBE2C	0	1.654258077	0.953	0.184	0
HMGB21	0	1.391234113	0.992	0.536	0
CKS21	0	1.240637008	0.994	0.56	0
H2AFZ1	0	1.157461646	1	0.961	0
HIST1H4C	0	1.11031714	0.925	0.659	0
CDK1	0	1.089758467	0.922	0.116	0
PLAU2	0	1.073253774	0.978	0.415	0
PCLAF1	0	1.061954541	0.998	0.571	0
TOP2A	0	1.058186921	0.892	0.139	0
CKS1B1	0	1.044099352	0.992	0.563	0
UBE2S1	0	1.013030551	0.999	0.833	0
STMN11	0	0.940151101	0.999	0.836	0
TUBA1B2	0	0.915851692	1	0.949	0
PTTG11	0	0.866600603	0.996	0.585	0
TYMS1	0	0.865318144	0.995	0.491	0
CENPF1	0	0.859670986	0.895	0.193	0
TUBB4B1	0	0.848706926	0.999	0.839	0
HMGN21	0	0.834878492	0.999	0.926	0
RANBP12	0	0.834760652	1	0.907	0
TMEM1583	0	0.818705985	0.999	0.851	0
MEST3	0	0.977290042	0.997	0.703	0
COL1A14	0	0.935296411	1	0.959	0
CCDC804	0	0.883088342	1	0.824	0
TPM12	0	0.840053401	1	0.942	0

SPARC2	0	0.767802906	1	0.96	0
COL1A23	0	0.762075954	1	0.962	0
SERPINE22	0	0.68241952	1	0.92	0
TAGLN3	0	0.659744507	1	0.959	0
ACTA22	0	0.636469774	0.973	0.702	0
COL3A13	0	0.602990184	1	0.949	0
ZFAS12	0	0.597499338	1	0.936	0
KRT183	0	0.573278905	0.984	0.783	0
CDH22	0	0.566469067	0.998	0.816	0
SULF11	0	0.561491731	0.947	0.504	0
RPS204	0	0.558580184	1	0.919	0
RPL13A4	0	0.539645565	1	0.959	0
DCN3	0	0.539456005	1	0.907	0
LOX1	0	0.520598552	1	0.858	0
IGFBP72	0	0.496381815	0.999	0.921	0
PTX33	7.89E-270	0.550518736	0.999	0.868	1.74E-265
LRRC75A	0	1.429942811	0.949	0.541	0
CLDN112	0	1.056826819	0.977	0.875	0
SCD2	0	0.84900618	0.954	0.86	0
PABPC13	0	0.82336043	0.991	0.934	0
EEF1A14	0	0.749393276	1	0.997	0
PHKG1	0	0.730737212	0.674	0.231	0
ADAM124	0	0.693340617	0.852	0.622	0
COL1A15	0	0.690846006	0.997	0.96	0
FTH13	0	0.63525491	1	1	0
EEF24	0	0.631549355	0.99	0.956	0
TTC3	0	0.616045255	0.973	0.92	0
DDR2	0	0.612308286	0.893	0.837	0
VCAN3	0	0.611440241	0.976	0.916	0
NEAT13	0	0.588407891	0.998	0.915	0
CCDC805	4.70E-276	0.669467133	0.966	0.828	1.04E-271
MALAT15	1.14E-265	0.638429021	0.999	0.962	2.51E-261
KCNQ1OT13	8.67E-263	0.701908313	0.821	0.664	1.91E-258
IGFBP34	3.35E-260	0.652486438	0.843	0.504	7.39E-256
MEST4	3.31E-254	0.625724829	0.904	0.71	7.30E-250
FLRT22	1.92E-219	0.5942429	0.732	0.564	4.23E-215
H2AFZ2	3.36E-195	0.684732914	0.884	0.967	7.41E-191
PRDX12	2.82E-182	0.67302288	0.81	0.967	6.22E-178
LDHA3	4.46E-182	0.789424706	0.781	0.958	9.84E-178
TFPI23	2.23E-138	1.025940891	0.793	0.86	4.92E-134
HSPE13	2.15E-104	0.699653749	0.733	0.955	4.73E-100
GSTO13	1.02E-82	0.681490548	0.695	0.959	2.26E-78
TUBB4B2	3.01E-45	0.817238528	0.641	0.858	6.65E-41
CKS22	3.12E-23	0.949688536	0.502	0.593	6.89E-19
MGST13	4.96E-22	0.66802234	0.617	0.91	1.10E-17
UBE2C1	9.55E-22	0.712453512	0.296	0.235	2.11E-17
MT-ND4L1	4.71E-15	0.78691767	0.416	0.863	1.04E-10
STMN12	1.04E-14	0.671807964	0.593	0.857	2.31E-10
FABP53	1.22E-14	0.740912665	0.573	0.86	2.70E-10
PLAU3	4.81E-13	0.669117797	0.44	0.455	1.06E-08
PTTG12	2.54E-09	0.757821175	0.479	0.618	5.61E-05
CXCL14	1.26E-08	0.776524018	0.449	0.53	0.000277549
ANGPTL44	7.78E-06	0.720100716	0.349	0.393	0.171710126
CCNB12	0.00011304	0.676505278	0.29	0.311	1
PSMA32	0.000289249	0.695145259	0.46	0.882	1
CTNNB11	0.002203711	0.733445141	0.431	0.785	1

STC14	0	1.082392187	0.943	0.343	0
PCLAF3	0	1.013728876	0.995	0.588	0
C12orf751	0	1.004854425	1	0.95	0
CAV13	0	0.923392355	1	0.932	0
STMN13	0	0.918315942	0.997	0.843	0
TM4SF13	0	0.905349801	0.977	0.543	0
HAS22	0	0.900914613	0.966	0.51	0
RRM23	0	0.894693986	0.977	0.266	0
HMGB23	0	0.882968652	0.97	0.554	0
UBE2C2	0	0.879456212	0.887	0.216	0
H2AFZ3	0	0.859573891	1	0.963	0
CKS23	0	0.851951162	0.979	0.577	0
ANLN2	0	0.805192774	0.93	0.193	0
TOP2A1	0	0.788778807	0.817	0.17	0
IFI271	0	0.78238968	0.7	0.215	0
MT2A5	0	0.767667435	1	0.997	0
ARL6IP13	0	0.762632489	0.935	0.635	0
ZWINT3	0	0.746138753	0.959	0.265	0
UBE2S3	0	0.742863107	0.997	0.839	0
MMP11	3.42E-230	0.761899377	0.451	0.147	7.56E-226
TIMP11	0	2.202839229	0.996	0.987	0
SERPINE12	0	1.427930046	0.842	0.9	0
CD631	0	1.045190761	0.95	0.985	0
MT2A6	0	0.966684294	0.999	0.997	0
IGFBP73	1.60E-225	1.474110333	0.771	0.93	3.52E-221
TFPI24	1.46E-168	1.12522688	0.813	0.859	3.23E-164
IFI63	4.42E-122	1.743295003	0.603	0.615	9.75E-118
CD93	1.04E-114	1.532084242	0.663	0.879	2.30E-110
PLOD21	2.59E-71	1.145187622	0.655	0.934	5.72E-67
ISG152	2.33E-61	1.390816225	0.604	0.831	5.15E-57
DCBLD23	3.02E-46	0.98506328	0.627	0.892	6.67E-42
MGST14	3.93E-40	1.002479098	0.606	0.91	8.67E-36
CYBA1	3.18E-33	1.027122622	0.578	0.887	7.02E-29
GAS61	2.50E-30	1.142736912	0.579	0.915	5.51E-26
GDF151	1.57E-19	1.015183459	0.314	0.639	3.46E-15
PLAT2	9.15E-15	0.94820205	0.57	0.883	2.02E-10
ANGPTL45	1.92E-13	1.192873974	0.358	0.393	4.24E-09
IFI272	6.84E-13	1.037749859	0.257	0.23	1.51E-08
REEP51	7.92E-07	1.00766171	0.434	0.89	0.017472222
CCND12	0.007791028	1.069336957	0.545	0.928	1
ISG153	0	1.574177774	0.976	0.821	0
CCND13	0	1.380950273	0.989	0.914	0
IFI64	0	1.3228104	0.938	0.607	0
TIMP12	0	1.288778371	1	0.987	0
MT2A7	0	1.210153084	1	0.997	0
C12orf753	0	1.178646345	0.997	0.951	0
CCND21	0	1.148135311	0.675	0.186	0
PHLDA25	0	1.095074896	0.985	0.889	0
S100A111	0	1.053142169	1	0.993	0
SELENOW4	0	1.005608926	0.993	0.938	0
MYL94	0	0.949351664	0.994	0.937	0
FKBP1A3	0	0.939662478	0.994	0.948	0
TSPO1	0	0.93056104	0.997	0.972	0
S100A101	0	0.909531415	1	0.983	0
SH3BGRL32	0	0.886307287	1	0.987	0
CHCHD102	7.93E-303	0.909574032	0.885	0.665	1.75E-298

ANXA22	2.50E-298	0.922444755	1	0.99	5.52E-294
TAGLN5	3.04E-267	0.969140298	0.998	0.96	6.72E-263
IFI273	1.23E-260	1.217507448	0.624	0.222	2.71E-256
BST21	1.25E-94	0.879893624	0.539	0.322	2.75E-90
HNRNPH14	4.01E-283	1.021469553	0.972	0.866	8.84E-279
HMGA15	8.50E-282	0.950372783	0.992	0.94	1.88E-277
TMEM1586	7.96E-220	0.96211423	0.983	0.859	1.76E-215
CTNNB15	4.17E-219	0.821213838	0.923	0.771	9.20E-215
SET2	1.13E-215	0.705872453	0.975	0.943	2.49E-211
MTDH3	1.08E-214	0.675700267	0.95	0.915	2.37E-210
MMP144	1.80E-212	0.848016462	0.909	0.824	3.97E-208
WNT5A6	4.90E-212	0.87928657	0.887	0.613	1.08E-207
STC15	1.48E-182	0.982971407	0.764	0.356	3.27E-178
ITGA26	2.36E-182	0.756649034	0.894	0.674	5.21E-178
HMGA23	9.05E-178	0.736310815	0.937	0.725	2.00E-173
CAPN25	1.10E-176	0.634234818	0.95	0.901	2.44E-172
GABBR23	5.60E-169	0.674767439	0.609	0.246	1.24E-164
PHLDA15	3.75E-158	0.693071226	0.971	0.84	8.28E-154
LRRC75A2	1.18E-155	0.740106296	0.806	0.553	2.60E-151
NRP14	1.89E-154	0.646190881	0.882	0.82	4.16E-150
NT5E5	1.80E-151	0.655652237	0.923	0.788	3.96E-147
PLAU5	2.81E-147	0.68813632	0.85	0.448	6.19E-143
B4GALT12	6.95E-146	0.66066507	0.839	0.768	1.53E-141
SACS4	3.93E-120	0.709608072	0.758	0.564	8.66E-116
GAPDH5	0	0.786693741	1	0.997	0
PPIA5	9.49E-263	0.782300241	0.998	0.982	2.09E-258
PFN17	2.28E-241	0.746379486	1	0.989	5.03E-237
EIF5A6	4.74E-205	0.767663011	0.99	0.949	1.05E-200
TPI16	1.06E-203	0.788315134	0.992	0.942	2.33E-199
PRDX15	6.13E-202	0.708161	0.993	0.962	1.35E-197
GSTO16	6.55E-191	0.728263306	0.978	0.949	1.45E-186
NME16	1.37E-180	0.716502549	0.98	0.927	3.02E-176
MT2A8	7.95E-177	0.869705699	1	0.997	1.76E-172
RANBP16	4.92E-174	0.858472355	0.977	0.912	1.09E-169
TAGLN27	8.16E-174	0.69422718	0.98	0.922	1.80E-169
FABP56	5.63E-134	0.712949222	0.94	0.849	1.24E-129
TFPI26	1.37E-128	1.145454637	0.975	0.856	3.02E-124
LDHA4	6.27E-126	0.656138354	0.97	0.952	1.38E-121
MT-ND4L4	6.63E-123	0.647224086	0.893	0.847	1.46E-118
HMGN24	4.45E-121	0.704932202	0.968	0.931	9.83E-117
PCLAF4	2.98E-120	0.79753588	0.865	0.598	6.57E-116
H2AFZ5	8.19E-111	0.703216175	0.988	0.964	1.81E-106
TYMS4	3.70E-80	0.677658615	0.728	0.523	8.16E-76
CXCL64	1.75E-61	0.758946065	0.768	0.6	3.87E-57
XIST2	2.35E-113	0.45667165	0.151	0.872	5.20E-109
RPL13A5	7.69E-67	0.635968729	0.91	0.962	1.70E-62
RPL27A5	5.11E-61	0.530686006	0.91	0.974	1.13E-56
TPM25	6.65E-61	0.566761845	0.92	0.991	1.47E-56
TAGLN6	6.97E-52	0.865343375	0.826	0.962	1.54E-47
TPM13	5.15E-50	0.937300865	0.788	0.946	1.14E-45
RPS205	3.78E-42	0.678936246	0.82	0.924	8.34E-38
HEXIM1	8.38E-40	0.505897543	0.063	0.397	1.85E-35
CALD14	3.48E-28	0.511271587	0.816	0.958	7.68E-24
KRT193	2.41E-23	0.492504533	0.222	0.589	5.31E-19
RPL316	2.69E-21	0.66869203	0.667	0.94	5.94E-17
CSRP13	2.15E-20	0.559022723	0.346	0.848	4.75E-16

MYL95	1.78E-15	0.679005638	0.654	0.941	3.92E-11
ANXA52	2.14E-13	0.457114624	0.686	0.966	4.73E-09
KRT84	8.06E-13	0.526131925	0.346	0.752	1.78E-08
ID32	4.73E-12	0.513214104	0.277	0.586	1.04E-07
RPS173	2.10E-09	0.471263258	0.417	0.898	4.63E-05
ANXA14	5.22E-05	0.487841472	0.627	0.953	1
IFITM24	0.005455545	0.537743856	0.486	0.901	1
KRT184	0.007997247	0.72649098	0.537	0.796	1
TOP2A3	3.84E-132	1.212766268	0.921	0.189	8.49E-128
NUSAP13	4.50E-132	0.876389014	0.907	0.192	9.94E-128
MKI674	1.15E-120	0.837003679	0.907	0.195	2.53E-116
CENPF4	1.23E-117	1.577156669	0.934	0.24	2.72E-113
CENPE3	1.83E-111	0.834856425	0.781	0.159	4.03E-107
TPX24	5.45E-104	1.04317484	0.954	0.284	1.20E-99
ASPM4	4.58E-99	0.885653774	0.907	0.233	1.01E-94
CCNB24	5.64E-89	0.739860917	0.901	0.258	1.24E-84
DEK4	1.13E-82	0.91459122	1	0.85	2.49E-78
NUCKS13	8.52E-77	0.846173053	1	0.919	1.88E-72
CKAP22	2.15E-75	0.699730847	0.874	0.307	4.75E-71
HMGB24	1.66E-71	1.138888238	0.993	0.566	3.66E-67
SMC44	6.34E-64	0.852680659	0.987	0.579	1.40E-59
HIST1H4C2	1.89E-60	1.563115609	0.993	0.677	4.17E-56
CCNB14	1.50E-57	0.881789503	0.848	0.308	3.31E-53
HMGB13	2.36E-53	0.754987065	1	0.949	5.22E-49
KPNA24	1.67E-49	0.81391548	0.96	0.608	3.70E-45
TUBA1C4	5.39E-47	0.737518892	1	0.906	1.19E-42
TUBA1B7	5.65E-43	0.752127008	1	0.953	1.25E-38
ACTA23	2.54E-25	0.700737923	0.947	0.715	5.61E-21
MEG32	2.43E-78	2.663054829	0.97	0.882	5.37E-74
NEAT14	7.34E-78	2.204999631	0.993	0.918	1.62E-73
F32	7.49E-59	2.337490407	0.866	0.522	1.65E-54
XIST3	5.83E-57	1.789795212	0.896	0.865	1.29E-52
SGIP11	6.38E-57	2.120688691	0.769	0.357	1.41E-52
MALAT17	1.48E-48	1.531095093	1	0.964	3.26E-44
MIR222HG5	1.63E-48	2.091077646	0.776	0.454	3.61E-44
VMP1	8.04E-46	1.996957178	0.858	0.904	1.77E-41
CCNL2	1.02E-39	1.898947172	0.769	0.568	2.25E-35
WSB11	1.75E-38	1.874939503	0.806	0.802	3.86E-34
NABP12	3.51E-38	2.130425439	0.754	0.541	7.76E-34
N4BP2L2	1.35E-37	1.711356728	0.806	0.827	2.99E-33
LENG81	1.85E-36	1.759674722	0.739	0.532	4.08E-32
HERC41	3.76E-36	1.696346289	0.769	0.641	8.29E-32
SLC17A9	1.65E-33	1.538188489	0.642	0.349	3.64E-29
AC016831.1	1.55E-26	1.523385584	0.567	0.303	3.42E-22
ACADVL	2.59E-24	1.538292423	0.724	0.718	5.71E-20
SLC4A72	7.19E-21	1.509176869	0.634	0.509	1.59E-16
ENC12	8.69E-20	1.507696975	0.59	0.404	1.92E-15
ARFGAP1	9.75E-11	1.544521607	0.515	0.447	2.15E-06

cluster
0
0
0
0
0
0
0
0
0
0
0
0
0
0
0
0
0
0
0
0
1
1
1
1
1
1
1
1
1
1
1
1
1
1
1
1
1
1
1
1
2
2
2
2
2
2
2
2
2
2
2
2

2
2
2
2
2
2
2
2
3
3
3
3
3
3
3
3
3
3
3
3
3
3
3
3
3
3
3
3
4
4
4
4
4
4
4
4
4
4
4
4
4
4
4
4
4
4
4
4
5
5
5
5
5
5
5
5

5
5
5
5
5
5
5
5
5
5
5
5
6
6
6
6
6
6
6
6
6
6
6
6
6
6
6
6
6
6
6
6
7
7
7
7
7
7
7
7
7
7
7
7
7
7
7
7
7
7
7
7
8
8
8
8

8
8
8
8
8
8
8
8
8
8
8
8
8
8
8
8
9
9
9
9
9
9
9
9
9
9
9
9
9
9
9
9
9
9
9
9
10
10
10
10
10
10
10
10
10
10
10
10
10
10
10
10
10
10
10
10

11
11
11
11
11
11
11
11
11
11
11
11
11
11
11
11
11
11
11
11
12
12
12
12
12
12
12
12
12
12
12
12
12
12
12
12
12
12
12
12
13
13
13
13
13
13
13
13
13
13
13
13
13
13
13
13

13
13
13
13
14
14
14
14
14
14
14
14
14
14
14
14
14
14
14
14
14
14
14
14
15
15
15
15
15
15
15
15
15
15
15
15
15
15
15
15
15
15
15
15
16
16
16
16
16
16
16
16
16
16
16
16

16
16
16
16
16
16
16
16
17
17
17
17
17
17
17
17
17
17
17
17
17
17
17
17
17
17
17
17
18
18
18
18
18
18
18
18
18
18
18
18
18
18
18
18
18
18
18
18

Supplementary Table 4. Immunoge

Gene	id
AC233755.1	ENSG00000275063
ACKR2	ENSG00000144648
ACOD1	ENSG00000102794
ADAR	ENSG00000160710
ADARB1	ENSG00000197381
ADGRB1	ENSG00000181790
ADGRE1	ENSG00000174837
ADGRE2	ENSG00000127507
AGER	ENSG00000204305
AIM2	ENSG00000163568
AIMP1	ENSG00000164022
AKAP8	ENSG00000105127
AKIRIN2	ENSG00000135334
ALCAM	ENSG00000170017
ALPK1	ENSG00000073331
ANKRD17	ENSG00000132466
ANXA1	ENSG00000135046
APOBEC3A	ENSG00000128383
APOBEC3B	ENSG00000179750
APOBEC3C	ENSG00000244509
APOBEC3D	ENSG00000243811
APOBEC3F	ENSG00000128394
APOBEC3G	ENSG00000239713
APOBEC3H	ENSG00000100298
ARG1	ENSG00000118520
ARG2	ENSG00000081181
ARHGEF2	ENSG00000116584
ARID5A	ENSG00000196843
ATG5	ENSG00000057663
ATRN	ENSG00000088812
AXL	ENSG00000167601
B2M	ENSG00000166710
BAG6	ENSG00000204463
BCL10	ENSG00000142867
BCL6	ENSG00000113916
BPIFA1	ENSG00000198183
BPIFB1	ENSG00000125999
BST2	ENSG00000130303
BTK	ENSG00000010671
BTLA	ENSG00000186265
BTN3A1	ENSG00000026950
BTN3A2	ENSG00000186470
BTN3A3	ENSG00000111801
BTNL8	ENSG00000113303
C17orf62	ENSG00000178927
C17orf99	ENSG00000187997
C1QA	ENSG00000173372
C1QB	ENSG00000173369
C1QBP	ENSG00000108561
C1QC	ENSG00000159189
C1R	ENSG00000159403
C1RL	ENSG00000139178

C1S	ENSG00000182326
C2	ENSG00000166278
C3	ENSG00000125730
C4A	ENSG00000244731
C4B	ENSG00000224389
C4BPA	ENSG00000123838
C4BPB	ENSG00000123843
C5	ENSG00000106804
C5orf30	ENSG00000181751
C6	ENSG00000039537
C6orf106	ENSG00000196821
C7	ENSG00000112936
C8A	ENSG00000157131
C8B	ENSG00000021852
C8G	ENSG00000176919
C9	ENSG00000113600
CACTIN	ENSG00000105298
CADM1	ENSG00000182985
CAMK1D	ENSG00000183049
CAMK4	ENSG00000152495
CARD9	ENSG00000187796
CASP4	ENSG00000196954
CCL11	ENSG00000172156
CCL13	ENSG00000181374
CCL16	ENSG00000275152
CCL17	ENSG00000102970
CCL18	ENSG00000275385
CCL19	ENSG00000172724
CCL2	ENSG00000108691
CCL20	ENSG00000115009
CCL21	ENSG00000137077
CCL23	ENSG00000274736
CCL24	ENSG00000106178
CCL25	ENSG00000131142
CCL26	ENSG00000006606
CCL3	ENSG00000277632
CCL4	ENSG00000275302
CCL4L2	ENSG00000276070
CCL5	ENSG00000271503
CCL7	ENSG00000108688
CCL8	ENSG00000108700
CCR2	ENSG00000121807
CD14	ENSG00000170458
CD160	ENSG00000117281
CD163	ENSG00000177575
CD180	ENSG00000134061
CD19	ENSG00000177455
CD1A	ENSG00000158477
CD1B	ENSG00000158485
CD1C	ENSG00000158481
CD1D	ENSG00000158473
CD1E	ENSG00000158488
CD209	ENSG00000090659
CD244	ENSG00000122223
CD247	ENSG00000198821
CD274	ENSG00000120217

CD300A	ENSG00000167851
CD300C	ENSG00000167850
CD300E	ENSG00000186407
CD300LB	ENSG00000178789
CD300LD	ENSG00000204345
CD300LF	ENSG00000186074
CD300LG	ENSG00000161649
CD3D	ENSG00000167286
CD3E	ENSG00000198851
CD3G	ENSG00000160654
CD4	ENSG00000010610
CD40	ENSG00000101017
CD46	ENSG00000117335
CD55	ENSG00000196352
CD5L	ENSG00000073754
CD6	ENSG00000013725
CD7	ENSG00000173762
CD74	ENSG00000019582
CD79A	ENSG00000105369
CD79B	ENSG00000007312
CD81	ENSG00000110651
CD84	ENSG00000066294
CD86	ENSG00000114013
CD8A	ENSG00000153563
CD8B	ENSG00000172116
CERS6	ENSG00000172292
CFB	ENSG00000243649
CFD	ENSG00000197766
CFH	ENSG00000000971
CFI	ENSG00000205403
CFP	ENSG00000126759
CGAS	ENSG00000164430
CHI3L1	ENSG00000133048
CHIA	ENSG00000134216
CHID1	ENSG00000177830
CHST1	ENSG00000175264
CHST2	ENSG00000175040
CHST4	ENSG00000140835
CLEC10A	ENSG00000132514
CLEC4A	ENSG00000111729
CLEC4C	ENSG00000198178
CLEC4D	ENSG00000166527
CLEC4E	ENSG00000166523
CLEC4M	ENSG00000104938
CLEC5A	ENSG00000258227
CLEC6A	ENSG00000205846
CLEC7A	ENSG00000172243
CLU	ENSG00000120885
CNPY3	ENSG00000137161
CNR2	ENSG00000188822
COLEC11	ENSG00000118004
CR1	ENSG00000203710
CR2	ENSG00000117322
CRACR2A	ENSG00000130038
CRCP	ENSG00000241258
CRTAM	ENSG00000109943

CSF1	ENSG00000184371
CSF1R	ENSG00000182578
CSK	ENSG00000103653
CTLA4	ENSG00000163599
CTPS1	ENSG00000171793
CXCL1	ENSG00000163739
CXCL10	ENSG00000169245
CXCL11	ENSG00000169248
CXCL13	ENSG00000156234
CXCL2	ENSG00000081041
CXCL3	ENSG00000163734
CXCL8	ENSG00000169429
CXCL9	ENSG00000138755
CYLD	ENSG00000083799
DAB2IP	ENSG00000136848
DBNL	ENSG00000136279
DCAF15	ENSG00000132017
DCLRE1C	ENSG00000152457
DCST1	ENSG00000163357
DCSTAMP	ENSG00000164935
DDX1	ENSG00000079785
DDX17	ENSG00000100201
DDX21	ENSG00000165732
DDX3X	ENSG00000215301
DDX58	ENSG00000107201
DDX60	ENSG00000137628
DHX36	ENSG00000174953
DHX58	ENSG00000108771
DHX9	ENSG00000135829
DTX3L	ENSG00000163840
ECSIT	ENSG00000130159
EIF2AK2	ENSG00000055332
EIF2AK4	ENSG00000128829
ELF3	ENSG00000163435
EOMES	ENSG00000163508
ERAP1	ENSG00000164307
ERAP2	ENSG00000164308
ETS1	ENSG00000134954
EXO1	ENSG00000174371
F2RL1	ENSG00000164251
FADD	ENSG00000168040
FBXO38	ENSG00000145868
FCAMR	ENSG00000162897
FCER1G	ENSG00000158869
FCGR1A	ENSG00000150337
FCGR1B	ENSG00000198019
FCGR2A	ENSG00000143226
FCMR	ENSG00000162894
FCN1	ENSG00000085265
FCN2	ENSG00000160339
FCN3	ENSG00000142748
FCRL4	ENSG00000163518
FFAR2	ENSG00000126262
FFAR3	ENSG00000185897
FFAR4	ENSG00000186188
FGA	ENSG00000171560

FGB	ENSG00000171564
FGL1	ENSG00000104760
FGR	ENSG00000000938
FYN	ENSG00000010810
G3BP1	ENSG00000145907
G3BP2	ENSG00000138757
GATA3	ENSG00000107485
GBP1	ENSG00000117228
GBP3	ENSG00000117226
GBP5	ENSG00000154451
GPER1	ENSG00000164850
GPR183	ENSG00000169508
GSDMD	ENSG00000104518
GZMM	ENSG00000197540
HAVCR2	ENSG00000135077
HCK	ENSG00000101336
HERC5	ENSG00000138646
HEXIM1	ENSG00000186834
HK1	ENSG00000156515
HLA-A	ENSG00000206503
HLA-B	ENSG00000234745
HLA-C	ENSG00000204525
HLA-DMA	ENSG00000204257
HLA-DMB	ENSG00000242574
HLA-DOA	ENSG00000204252
HLA-DOB	ENSG00000241106
HLA-DPA1	ENSG00000231389
HLA-DPB1	ENSG00000223865
HLA-DQA1	ENSG00000196735
HLA-DQA2	ENSG00000237541
HLA-DQB2	ENSG00000232629
HLA-DRA	ENSG00000204287
HLA-DRB1	ENSG00000196126
HLA-DRB5	ENSG00000198502
HLA-F	ENSG00000204642
HLA-G	ENSG00000204632
HMGB1	ENSG00000189403
HMGB2	ENSG00000164104
HMGB3	ENSG00000029993
HMHB1	ENSG00000158497
HP	ENSG00000257017
ICOSLG	ENSG00000160223
IDO1	ENSG00000131203
IDO2	ENSG00000188676
IFI16	ENSG00000163565
IFI30	ENSG00000216490
IFI6	ENSG00000126709
IFIH1	ENSG00000115267
IFIT1	ENSG00000185745
IFIT2	ENSG00000119922
IFIT3	ENSG00000119917
IFIT5	ENSG00000152778
IFITM1	ENSG00000185885
IFITM2	ENSG00000185201
IFITM3	ENSG00000142089
IL17C	ENSG00000124391

IL17RE	ENSG00000163701
IL18	ENSG00000150782
IL18R1	ENSG00000115604
IL18RAP	ENSG00000115607
IL1A	ENSG00000115008
IL1B	ENSG00000125538
IL1R1	ENSG00000115594
IL1RAP	ENSG00000196083
IL1RL2	ENSG00000115598
IL2	ENSG00000109471
IL23A	ENSG00000110944
IL23R	ENSG00000162594
IL27	ENSG00000197272
IL2RA	ENSG00000134460
IL31	ENSG00000204671
IL31RA	ENSG00000164509
IL34	ENSG00000157368
IL36A	ENSG00000136694
IL36B	ENSG00000136696
IL36G	ENSG00000136688
IL36RN	ENSG00000136695
IL4R	ENSG00000077238
INAVA	ENSG00000163362
INPP5D	ENSG00000168918
INPPL1	ENSG00000165458
IRAK1	ENSG00000184216
IRAK4	ENSG00000198001
IRF1	ENSG00000125347
IRF3	ENSG00000126456
IRF5	ENSG00000128604
IRF7	ENSG00000185507
IRGM	ENSG00000237693
ISG15	ENSG00000187608
ISG20	ENSG00000172183
ITCH	ENSG00000078747
ITGAM	ENSG00000169896
ITK	ENSG00000113263
JAGN1	ENSG00000171135
JAK2	ENSG00000096968
JAK3	ENSG00000105639
JAML	ENSG00000160593
KCNN4	ENSG00000104783
KDM6B	ENSG00000132510
KLKB1	ENSG00000164344
KLRG1	ENSG00000139187
KLRK1	ENSG00000213809
KNG1	ENSG00000113889
LACC1	ENSG00000179630
LAG3	ENSG00000089692
LAIR1	ENSG00000167613
LAMP3	ENSG00000078081
LAT	ENSG00000213658
LAT2	ENSG00000086730
LAX1	ENSG00000122188
LBP	ENSG00000129988
LCN2	ENSG00000148346

LGALS3	ENSG00000131981
LGALS9	ENSG00000168961
LGR4	ENSG00000205213
LILRA1	ENSG00000104974
LILRA2	ENSG00000239998
LILRA4	ENSG00000239961
LILRA5	ENSG00000187116
LILRA6	ENSG00000244482
LILRB2	ENSG00000131042
LILRB3	ENSG00000204577
LILRB4	ENSG00000186818
LILRB5	ENSG00000105609
LIME1	ENSG00000203896
LOXL3	ENSG00000115318
LRMP	ENSG00000118308
LST1	ENSG00000204482
LTF	ENSG00000012223
LXN	ENSG00000079257
LY86	ENSG00000112799
LY9	ENSG00000122224
LY96	ENSG00000154589
LYAR	ENSG00000145220
LYN	ENSG00000254087
MAP3K5	ENSG00000197442
MAP3K8	ENSG00000107968
MAP4K2	ENSG00000168067
MARCO	ENSG00000019169
MASP1	ENSG00000127241
MASP2	ENSG00000009724
MATR3	ENSG00000015479
MAVS	ENSG00000088888
MBL2	ENSG00000165471
MCM3AP	ENSG00000160294
MCOLN1	ENSG00000090674
MCOLN2	ENSG00000153898
MEFV	ENSG00000103313
MEP1B	ENSG00000141434
METTL3	ENSG00000165819
MFHAS1	ENSG00000147324
MICB	ENSG00000204516
MIF	ENSG00000240972
MPEG1	ENSG00000197629
MR1	ENSG00000153029
MSMP	ENSG00000215183
MSRB1	ENSG00000198736
MST1R	ENSG00000164078
MX1	ENSG00000157601
MX2	ENSG00000183486
MYD88	ENSG00000172936
MYO1G	ENSG00000136286
NAIP	ENSG00000249437
NCR3	ENSG00000204475
NDST1	ENSG00000070614
NFKBID	ENSG00000167604
NLRC4	ENSG00000091106
NLRC5	ENSG00000140853

NLRP1	ENSG00000091592
NLRP10	ENSG00000182261
NLRP2	ENSG00000022556
NLRP2B	ENSG00000215174
NLRP3	ENSG00000162711
NLRP4	ENSG00000160505
NLRP6	ENSG00000174885
NLRP9	ENSG00000185792
NLRX1	ENSG00000160703
NOD1	ENSG00000106100
NOD2	ENSG00000167207
NONO	ENSG00000147140
NR1H4	ENSG00000012504
NUDCD1	ENSG00000120526
OAS1	ENSG00000089127
OAS2	ENSG00000111335
OAS3	ENSG00000111331
OASL	ENSG00000135114
OLR1	ENSG00000173391
OPTN	ENSG00000123240
ORAI1	ENSG00000276045
OTUB1	ENSG00000167770
OTUD4	ENSG00000164164
OTUD7B	ENSG00000264522
OTULIN	ENSG00000154124
PADI4	ENSG00000159339
PAG1	ENSG00000076641
PARK7	ENSG00000116288
PARP14	ENSG00000173193
PARP9	ENSG00000138496
PCBP2	ENSG00000197111
PDCD1	ENSG00000188389
PDCD1LG2	ENSG00000197646
PGLYRP1	ENSG00000008438
PGLYRP2	ENSG00000161031
PGLYRP3	ENSG00000159527
PGLYRP4	ENSG00000163218
PIBF1	ENSG00000083535
PIK3CD	ENSG00000171608
PIK3CG	ENSG00000105851
PLA2G2A	ENSG00000188257
PLA2G2E	ENSG00000188784
PLA2G2F	ENSG00000158786
PLD3	ENSG00000105223
PLD4	ENSG00000166428
PLGRKT	ENSG00000107020
PML	ENSG00000140464
POLR3A	ENSG00000148606
POLR3B	ENSG00000013503
POLR3C	ENSG00000186141
POLR3D	ENSG00000168495
POLR3E	ENSG00000058600
POLR3F	ENSG00000132664
POLR3G	ENSG00000113356
POLR3H	ENSG00000100413
POLR3K	ENSG00000161980

PPP6C	ENSG00000119414
PQBP1	ENSG00000102103
PRDM1	ENSG00000057657
PRG2	ENSG00000186652
PRKCB	ENSG00000166501
PRKCE	ENSG00000171132
PRKCQ	ENSG00000065675
PRKCZ	ENSG00000067606
PRKD1	ENSG00000184304
PRKD2	ENSG00000105287
PRKDC	ENSG00000253729
PRR7	ENSG00000131188
PSMA1	ENSG00000129084
PSMB8	ENSG00000204264
PSMB9	ENSG00000240065
PSPC1	ENSG00000121390
PSTPIP1	ENSG00000140368
PTK2B	ENSG00000120899
PTMS	ENSG00000159335
PTPN22	ENSG00000134242
PYCARD	ENSG00000103490
PYDC2	ENSG00000253548
RAET1E	ENSG00000164520
RAET1G	ENSG00000203722
RAET1L	ENSG00000155918
RARRES2	ENSG00000106538
RBM14	ENSG00000239306
REG3A	ENSG00000172016
REG3G	ENSG00000143954
RIOK3	ENSG00000101782
RIPK1	ENSG00000137275
RIPK2	ENSG00000104312
RNASET2	ENSG00000026297
RNF125	ENSG00000101695
RNF135	ENSG00000181481
RNF166	ENSG00000158717
RNF19B	ENSG00000116514
RPS6KA4	ENSG00000162302
RPS6KA5	ENSG00000100784
RSAD2	ENSG00000134321
RUBCN	ENSG00000145016
S100A12	ENSG00000163221
S100A8	ENSG00000143546
S100A9	ENSG00000163220
SAMHD1	ENSG00000101347
SARM1	ENSG00000004139
SCART1	ENSG00000214279
SEC14L1	ENSG00000129657
SEMA4A	ENSG00000196189
SEMA7A	ENSG00000138623
SERINC3	ENSG00000132824
SERINC5	ENSG00000164300
SERPING1	ENSG00000149131
SETD2	ENSG00000181555
SFPQ	ENSG00000116560
SFTPD	ENSG00000133661

SH2D1A	ENSG00000183918
SH2D1B	ENSG00000198574
SIGLEC10	ENSG00000142512
SIRT2	ENSG00000068903
SIT1	ENSG00000137078
SKAP1	ENSG00000141293
SKP2	ENSG00000145604
SLAMF1	ENSG00000117090
SLAMF6	ENSG00000162739
SLAMF7	ENSG00000026751
SLFN11	ENSG00000172716
SLPI	ENSG00000124107
SMPDL3B	ENSG00000130768
SPON2	ENSG00000159674
SQSTM1	ENSG00000161011
SRC	ENSG00000197122
SSC5D	ENSG00000179954
STAB1	ENSG00000010327
STMP1	ENSG00000243317
SUSD4	ENSG00000143502
SYK	ENSG00000165025
TAP1	ENSG00000168394
TAP2	ENSG00000204267
TAPBPL	ENSG00000139192
TARM1	ENSG00000248385
TBK1	ENSG00000183735
TBKBP1	ENSG00000198933
TEC	ENSG00000135605
TFE3	ENSG00000068323
TFEB	ENSG00000112561
THEMIS	ENSG00000172673
THEMIS2	ENSG00000130775
TICAM1	ENSG00000127666
TICAM2	ENSG00000243414
TIFA	ENSG00000145365
TIRAP	ENSG00000150455
TLR1	ENSG00000174125
TLR10	ENSG00000174123
TLR2	ENSG00000137462
TLR3	ENSG00000164342
TLR4	ENSG00000136869
TLR5	ENSG00000187554
TLR6	ENSG00000174130
TLR7	ENSG00000196664
TLR8	ENSG00000101916
TLR9	ENSG00000239732
TMEM106A	ENSG00000184988
TMEM173	ENSG00000184584
TNFAIP3	ENSG00000118503
TNFAIP8L2	ENSG00000163154
TNFRSF13B	ENSG00000240505
TNFRSF13C	ENSG00000159958
TNFRSF14	ENSG00000157873
TNFRSF17	ENSG00000048462
TNFRSF21	ENSG00000146072
TNFSF13	ENSG00000161955

TNFSF13B	ENSG00000102524
TNFSF18	ENSG00000120337
TNIP1	ENSG00000145901
TNIP2	ENSG00000168884
TNIP3	ENSG00000050730
TOLLIP	ENSG00000078902
TRAF3	ENSG00000131323
TRAF6	ENSG00000175104
TRAT1	ENSG00000163519
TREML4	ENSG00000188056
TRIL	ENSG00000255690
TRIM13	ENSG00000204977
TRIM14	ENSG00000106785
TRIM23	ENSG00000113595
TRIM25	ENSG00000121060
TRIM26	ENSG00000234127
TRIM29	ENSG00000137699
TRIM38	ENSG00000112343
TRIM4	ENSG00000146833
TRIM5	ENSG00000132256
TRIM56	ENSG00000169871
TRIM8	ENSG00000171206
TRPM4	ENSG00000130529
TTC4	ENSG00000243725
TTLL12	ENSG00000100304
TXK	ENSG00000074966
TYROBP	ENSG00000011600
ULBP1	ENSG00000111981
ULBP2	ENSG00000131015
ULBP3	ENSG00000131019
UNC93B1	ENSG00000110057
USP14	ENSG00000101557
VSIG4	ENSG00000155659
VSTM1	ENSG00000189068
VTCN1	ENSG00000134258
WRNIP1	ENSG00000124535
XRCC5	ENSG00000079246
XRCC6	ENSG00000196419
YTHDF1	ENSG00000149658
YTHDF2	ENSG00000198492
ZAP70	ENSG00000115085
ZBP1	ENSG00000124256
ZBTB1	ENSG00000126804
ZC3H12A	ENSG00000163874
ZC3HAV1	ENSG00000105939
ZCCHC3	ENSG00000247315
ZNF580	ENSG00000213015
ZNF683	ENSG00000176083

Supplementary Table 5. DEGs of CD317posi_vs_nega

	avg_log2FC	p_val_adj	p_val
BST2	2.678203883	0	0
NEAT1	1.335093895	0	0
IGFBP5	1.323527798	0	0
COL1A1	1.266654098	0	0
MALAT1	1.236918778	0	0
ITGB1	1.103073879	0	0
MT-CO1	1.058827116	0	0
MT-CO2	1.003593334	0	0
FN1	0.981099371	0	0
CCDC80	0.973012066	0	0
MT-CO3	0.953996611	0	0
COL6A1	0.946147952	0	0
MT-CYB	0.94450672	0	0
MT-ND1	0.921610959	0	0
PLOD2	0.920521196	0	0
PTX3	0.918334049	0	0
COL1A2	0.909975418	0	0
RTN4	0.894564234	0	0
IFI6	0.872412928	0	0
MT-ND3	0.86166079	0	0
MT-ND4	0.846827637	0	0
SPARC	0.843551482	0	0
MT-ND2	0.836730586	0	0
CKAP4	0.824366141	0	0
TGFBI	0.813754343	0	0
THBS1	0.811191158	0	0
COL3A1	0.810782745	0	0
VCAN	0.796652212	0	0
B2M	0.794065831	0	0
COL6A3	0.793890381	0	0
ATP2B1	0.793382435	0	0
HSP90B1	0.778472587	0	0
CALU	0.773286183	0	0
CLU	0.76255402	0	0
LUM	0.762118738	0	0
HLA-C	0.759416248	0	0
PLAT	0.750881009	0	0
DCN	0.737440735	0	0
RRBP1	0.73457364	0	0
TIMP3	0.733077497	0	0
COL6A2	0.729987992	0	0
LOX	0.729285645	0	0
COL5A2	0.722953408	0	0
HLA-A	0.719456978	0	0
CDH2	0.704843081	0	0
IGFBP3	0.704471961	0	0
MT-ATP6	0.703423632	0	0
HLA-B	0.70227463	0	0
MME	0.699257286	0	0
CD81	0.695471169	0	0
NRP2	0.693935383	0	0
CTGF	0.687215511	0	0
APP	0.686491809	0	0

HSPA5	0.683871264	0	0
MT-ND5	0.67297582	0	0
SPOCK1	0.67102491	0	0
CLDN11	0.663902579	0	0
SERPINE1	0.658345069	0	0
MDK	0.655746805	0	0
MAP1B	0.655356679	0	0
DCBLD2	0.655308774	0	0
MEST	0.650586739	0	0
LMAN1	0.645198302	0	0
CTSB	0.639826168	0	0
PXDN	0.63701997	0	0
LRP1	0.631211271	0	0
SEC62	0.63015989	0	0
THY1	0.627472214	0	0
SCD	0.626882994	0	0
GREM1	0.622867489	0	0
CYR61	0.621423941	0	0
COL12A1	0.619312574	0	0
AHNAK	0.616056547	0	0
MEG3	0.609101682	0	0
MMP2	0.607868356	0	0
CCND1	0.607269932	0	0
POSTN	0.607124092	0	0
PAPPA	0.607060434	0	0
TIMP1	0.60682235	0	0
KCNQ1OT1	0.606129002	0	0
CALR	0.605114172	0	0
IL6ST	0.601631841	0	0
IFI27	0.601392369	0	0
SERPINH1	0.601327569	0	0
CALD1	0.597022042	0	0
OLFML3	0.595365555	0	0
DST	0.592495501	0	0
COL8A1	0.588985536	0	0
NPC2	0.586605842	0	0
PPIB	0.586033648	0	0
IGFBP6	0.585271801	0	0
PSAP	0.585010534	0	0
P4HA1	0.574942617	0	0
IGFBP7	0.573828307	0	0
MYADM	0.570902472	0	0
GRN	0.570725643	0	0
VCL	0.569414179	0	0
TMBIM6	0.565863499	0	0
MFGE8	0.565179899	0	0
P4HB	0.564269505	0	0
LY6E	0.56098725	0	0
ITM2B	0.556474289	0	0
CD63	0.551585173	0	0
SLC20A1	0.551251205	0	0
FSTL1	0.550976215	0	0
CANX	0.547365502	0	0
ZFP36L1	0.542782667	0	0
ATP6AP2	0.541541985	0	0
P4HA2	0.541301904	0	0

RHOBTB3	0.540612205	0	0
CHI3L1	0.536394922	0	0
LAPTM4A	0.535810301	0	0
MTDH	0.532673445	0	0
COL5A1	0.532325534	0	0
PTTG1IP	0.53218057	0	0
MACF1	0.531811739	0	0
ADAM9	0.530949654	0	0
COL4A2	0.529961925	0	0
TGOLN2	0.529150899	0	0
CD164	0.526966625	0	0
LGALS3BP	0.525779096	0	0
KTN1	0.524560023	0	0
FBN1	0.52307043	0	0
SPTBN1	0.523033026	0	0
LAMP1	0.520621027	0	0
APLP2	0.51883883	0	0
CD9	0.518299575	0	0
BSG	0.517621512	0	0
XIST	0.517085157	0	0
FBLN1	0.516948097	0	0
CDH11	0.516443018	0	0
CTSA	0.514488279	0	0
LRPAP1	0.514177486	0	0
MRC2	0.513092988	0	0
PDIA6	0.512568519	0	0
LOXL2	0.512429917	0	0
DDR2	0.505736906	0	0
UGCG	0.497771081	0	0
PDGFRA	0.497696813	0	0
CST3	0.497663714	0	0
INHBA	0.497390402	0	0
PDIA3	0.497211311	0	0
FADS1	0.496340665	0	0
RCN1	0.494924677	0	0
SERINC1	0.493560038	0	0
ARL6IP5	0.490675584	0	0
TRAM1	0.485844656	0	0
SQLE	0.48479232	0	0
SERPINB2	0.483671237	0	0
RPN2	0.481290448	0	0
TIMP2	0.480296155	0	0
CD44	0.477359698	0	0
FRMD6	0.475334912	0	0
PRNP	0.47514062	0	0
CYP1B1	0.473352616	0	0
CYBA	0.471063518	0	0
IQGAP1	0.468463077	0	0
TMED10	0.467046498	0	0
GJA1	0.463986018	0	0
SSR4	0.463700467	0	0
CRIM1	0.463138371	0	0
TLN1	0.458166828	0	0
LEPROT	0.456613086	0	0
FLNA	0.455860784	0	0
CD99	0.45360788	0	0

KDELR1	0.452471899	0	0
TGFBR2	0.451379715	0	0
EIF3A	0.45126186	0	0
GAS6	0.449327001	0	0
NUCB2	0.449268183	0	0
ASPH	0.448762559	0	0
F3	0.446854445	0	0
PCOLCE	0.445719001	0	0
CTSD	0.439425575	0	0
GLG1	0.438805367	0	0
SERPINE2	0.437823685	0	0
QSOX1	0.437817412	0	0
ERP29	0.437140102	0	0
GANAB	0.435211489	0	0
DKK1	0.434901032	0	0
EXT1	0.433449421	0	0
PPIC	0.43054839	0	0
LAMC1	0.430472901	0	0
TM9SF3	0.429092808	0	0
ADAM12	0.423499849	0	0
FKBP10	0.423419977	0	0
LAMP2	0.423230245	0	0
ACSL3	0.423073174	0	0
PRRC2C	0.421784948	0	0
CD59	0.421273729	0	0
TPBG	0.415284021	0	0
PLD3	0.412144111	0	0
GPNMB	0.411936434	0	0
DDOST	0.410566308	0	0
DAB2	0.40909482	0	0
CLMP	0.408052	0	0
LRP10	0.406369861	0	0
SLC38A2	0.405577493	0	0
RGS4	0.405054321	0	0
SEC61A1	0.404119535	0	0
NT5E	0.40358889	0	0
SERINC3	0.401594333	0	0
ALCAM	0.400678456	0	0
NRP1	0.400582993	0	0
ANKRD11	0.399614323	0	0
PLEC	0.398988459	0	0
LRRC17	0.398203018	0	0
MAN1A1	0.397535876	0	0
TMED9	0.395959554	0	0
MYH9	0.393428006	0	0
CADM1	0.39330229	0	0
TMEM59	0.393110973	0	0
C1R	0.392952071	0	0
PNISR	0.392503005	0	0
MAN2A1	0.392353258	0	0
EMILIN1	0.390969926	0	0
COL4A1	0.389622873	0	0
CD151	0.389298526	0	0
DPP4	0.388365939	0	0
SPON2	0.386126723	0	0
CCL2	0.386125664	0	0

NOTCH2	0.386016397	0	0
STAT1	0.385794663	0	0
CCND2	0.382420331	0	0
LIMA1	0.381898614	0	0
MCFD2	0.380924801	0	0
GOLGA4	0.379641959	0	0
SOX4	0.377494665	0	0
CRTAP	0.3768918	0	0
MSMO1	0.375275781	0	0
AKAP12	0.373693541	0	0
C1S	0.373457263	0	0
DKK3	0.372041985	0	0
ACTN1	0.372028686	0	0
BGN	0.371632199	0	0
PCYOX1	0.37086808	0	0
ANTXR2	0.369723628	0	0
PTN	0.368226603	0	0
CHPF	0.368203479	0	0
VMP1	0.36762641	0	0
SEMA3C	0.36577115	0	0
SLC38A1	0.364961327	0	0
ITM2C	0.363964233	0	0
GOLGB1	0.363692776	0	0
SEC63	0.362106821	0	0
HEG1	0.361310429	0	0
ISG15	0.361275896	0	0
FAP	0.361051819	0	0
HSD17B12	0.36067459	0	0
MYDGF	0.359981709	0	0
MAP4K4	0.35877202	0	0
PLOD1	0.3577294	0	0
MXRA8	0.35697527	0	0
TMCO1	0.356812195	0	0
THBS2	0.354871714	0	0
KPNB1	0.351431036	0	0
NID2	0.351117035	0	0
AKAP9	0.350722873	0	0
TMEM200A	0.350342263	0	0
PDIA4	0.350035568	0	0
ASAP1	0.349637445	0	0
HMGA2	0.349209368	0	0
CREB3L1	0.348101107	0	0
CDKN1A	0.34782854	0	0
ITGA5	0.347812335	0	0
PERP	0.347637393	0	0
SSR1	0.345899388	0	0
CD46	0.345613739	0	0
TTC3	0.344507668	0	0
DPYSL2	0.344388703	0	0
SLC3A2	0.344128189	0	0
DYNC1H1	0.344033589	0	0
CCPG1	0.343814538	0	0
SMURF2	0.343352466	0	0
FLRT2	0.341955792	0	0
11-Sep	0.340113385	0	0
UACA	0.339645789	0	0

KLF6	0.339152968	0	0
FADS2	0.338997046	0	0
OSBPL8	0.338746244	0	0
OS9	0.33841385	0	0
FAM118A	0.337383524	0	0
ATP6V0E1	0.337010513	0	0
DHRS3	0.33540474	0	0
ATRX	0.335350421	0	0
VKORC1	0.335229528	0	0
SLC5A3	0.334726694	0	0
MARCKS	0.333633956	0	0
FNDC3B	0.333518345	0	0
GLIPR1	0.333338287	0	0
XBP1	0.333319698	0	0
YIPF5	0.332973625	0	0
TRAM2	0.332863605	0	0
UBXN4	0.332751238	0	0
LPP	0.332044902	0	0
RPN1	0.331695952	0	0
ARID5B	0.331191223	0	0
DSG2	0.331031786	0	0
TPP1	0.330782295	0	0
PALLD	0.330626794	0	0
ITGA2	0.330547763	0	0
NFE2L1	0.330051221	0	0
RAI14	0.329793401	0	0
PABPC1	0.329509984	0	0
LDLR	0.327661038	0	0
RABAC1	0.327550108	0	0
ADAM10	0.327162681	0	0
EDIL3	0.327121665	0	0
ZMAT3	0.326879751	0	0
TAPBP	0.325640107	0	0
CD248	0.325059537	0	0
LTBP2	0.32293299	0	0
ITGAV	0.322810371	0	0
CDC42EP3	0.322795712	0	0
DHCR24	0.321306247	0	0
MMP14	0.321300036	0	0
DDAH1	0.320537339	0	0
SON	0.320391755	0	0
LAMB1	0.319849532	0	0
SLC1A5	0.319198719	0	0
RTN3	0.318822768	0	0
SERPINF1	0.317132213	0	0
STC2	0.316138608	0	0
HLA-E	0.315175111	0	0
TFRC	0.31459485	0	0
ATP2B4	0.312808674	0	0
EMC7	0.312581437	0	0
POLR2J3.1	0.312317302	0	0
SEC31A	0.31072893	0	0
NUCB1	0.308661378	0	0
PRSS12	0.308606226	0	0
ATP6AP1	0.307447301	0	0
IFITM3	0.305620129	0	0

PLOD3	0.305429997	0	0
CYBRD1	0.304825129	0	0
FILIP1L	0.304512201	0	0
SELENOT	0.30418435	0	0
FCGRT	0.303996563	0	0
GDF15	0.303821293	0	0
ADAMTS1	0.30332407	0	0
NPTN	0.303145453	0	0
GRINA	0.302317241	0	0
TXNIP	0.302162969	0	0
PGRMC2	0.301919067	0	0
FXYD5	0.301062865	0	0
PRDX4	0.300796906	0	0
SULF1	0.300516808	0	0
COPA	0.300164351	0	0
FGF7	0.299378965	0	0
ZEB1	0.298861529	0	0
MESD	0.298218976	0	0
NTM	0.297926108	0	0
SCARB2	0.297285376	0	0
SRRM2	0.296720185	0	0
DDX17	0.296664039	0	0
GAS1	0.296574504	0	0
TMEM30A	0.295904041	0	0
INSIG1	0.29570081	0	0
ECM1	0.295690177	0	0
IGF2R	0.295553071	0	0
EGFR	0.295167757	0	0
DNAJC1	0.295113093	0	0
PRKCSH	0.294907698	0	0
NR2F2	0.293932768	0	0
COL14A1	0.293845231	0	0
GNS	0.293821157	0	0
CAP1	0.293389162	0	0
ERGIC3	0.292783229	0	0
FLNB	0.29228729	0	0
FBN2	0.291904214	0	0
ABCA1	0.291613981	0	0
CPA4	0.291603047	0	0
ENC1	0.291393629	0	0
MLEC	0.290622346	0	0
MYOF	0.290378933	0	0
ITGB5	0.290345308	0	0
CASC4	0.289764102	0	0
CDH13	0.289618026	0	0
HEXB	0.287185828	0	0
JAK1	0.286741864	0	0
AXL	0.286174636	0	0
FLT1	0.285895234	0	0
SSR2	0.285689908	0	0
FKBP14	0.284321739	0	0
TCAF1	0.283978396	0	0
ZNF106	0.283515316	0	0
LPAR1	0.282557099	0	0
LMAN2	0.282475742	0	0
SSFA2	0.282013415	0	0

ASAH1	0.281828256	0	0
SEL1L	0.281030447	0	0
WSB1	0.279717588	0	0
FGFR1	0.279197755	0	0
SIL1	0.278874937	0	0
SELENOS	0.27879503	0	0
NEXN	0.278336397	0	0
TMEM165	0.278237535	0	0
LMO7	0.278222825	0	0
RCN3	0.27805324	0	0
CLTC	0.276879675	0	0
SURF4	0.276354007	0	0
SOD2	0.276168938	0	0
ABL2	0.276140732	0	0
P3H1	0.274930627	0	0
DSE	0.274497162	0	0
ADD3	0.274484043	0	0
SPTAN1	0.274127862	0	0
TGM2	0.273034312	0	0
SGIP1	0.272789004	0	0
ATP2A2	0.272659632	0	0
CTSZ	0.272389887	0	0
SELENOM	0.271880797	0	0
SUMF2	0.271335954	0	0
JCAD	0.27041593	0	0
WLS	0.270063522	0	0
DHCR7	0.269618708	0	0
ERLEC1	0.269319536	0	0
TPR	0.269147009	0	0
YWHAG	0.267995677	0	0
MAGT1	0.26774366	0	0
REEP5	0.267368079	0	0
ITGA11	0.267244032	0	0
HEXA	0.26681626	0	0
TWSG1	0.266608342	0	0
SLC16A3	0.266215021	0	0
MMP1	0.266022456	0	0
FBLN5	0.26524982	0	0
MAP1A	0.26507068	0	0
HOXC8	0.263824622	0	0
ANTXR1	0.26300161	0	0
ADAMTS6	0.262905453	0	0
FKBP9	0.261630601	0	0
EFEMP2	0.261602472	0	0
DEGS1	0.260848513	0	0
GLS	0.260719733	0	0
COL11A1	0.260474782	0	0
SDC4	0.25991095	0	0
NR3C1	0.259503274	0	0
USO1	0.259280102	0	0
MYCBP2	0.259061999	0	0
PCNX4	0.259055803	0	0
SLC39A6	0.258649439	0	0
AAK1	0.257885754	0	0
FAM120A	0.257751377	0	0
HERPUD1	0.257690416	0	0

HTRA1	0.257206453	0	0
MEIS2	0.257071191	0	0
RGMB	0.25706933	0	0
TMED7	0.256861741	0	0
CDCP1	0.256786363	0	0
ECE1	0.256749573	0	0
DPP7	0.256203193	0	0
STT3B	0.255873953	0	0
ACTR2	0.2549185	0	0
GALNT2	0.254805338	0	0
EREG	0.254626062	0	0
CTSK	0.254530793	0	0
SCPEP1	0.253744698	0	0
RBMS1	0.253471236	0	0
ELL2	0.25319223	0	0
PRAF2	0.253163225	0	0
ICAM1	0.252155781	0	0
CDK6	0.251889232	0	0
ENAH	0.251789423	0	0
C12orf75	0.251674661	0	0
EMC10	0.251471621	0	0
GALNT1	0.251363157	0	0
TM9SF2	0.251314201	0	0
EEA1	0.251047319	0	0
GTF2I	0.251038751	0	0
ATP2C1	0.250963622	0	0
LTBP1	0.250476275	0	0
ARHGAP29	0.250437021	0	0
ARMCX3	0.250206107	0	0
CMTM6	0.250185566	0	0
ELOC	-0.250078749	0	0
DDT	-0.250483685	0	0
CARD19	-0.250526661	0	0
CCNC	-0.250656571	0	0
TOMM22	-0.250858547	0	0
NDUFS4	-0.251132456	0	0
IMP3	-0.251389822	0	0
EIF4A3	-0.251699262	0	0
FKBP3	-0.25203337	0	0
TMEM167A	-0.252135497	0	0
SRM	-0.252303015	0	0
ATP5F1D	-0.252445276	0	0
ATP5PO	-0.252524522	0	0
JUNB	-0.252749864	0	0
MTCH2	-0.252758314	0	0
SSNA1	-0.253272294	0	0
COMMD8	-0.254204767	0	0
ODC1	-0.254382481	0	0
TATDN1	-0.254399222	0	0
PIN1	-0.254545973	0	0
MPHOSPH6	-0.254625232	0	0
ATP5MF	-0.254813255	0	0
EIF3K	-0.2549175	0	0
SNHG25	-0.255218509	0	0
MIF	-0.255346383	0	0
RPL29	-0.25554562	3.31E-98	1.49E-102

NUP37	-0.256585654	0	0
MPLKIP	-0.256828282	0	0
COA6	-0.256892261	0	0
SRSF5	-0.257003233	0	0
MRFAP1	-0.257661637	0	0
RPL18A	-0.257848348	1.63E-73	7.35E-78
PSMD13	-0.257851222	0	0
PSMG2	-0.25787911	0	0
EMC6	-0.257890837	0	0
TEX30	-0.258421183	0	0
COMMD4	-0.258447456	0	0
MRPL19	-0.259281089	0	0
DRAP1	-0.259337134	0	0
HYI	-0.260020126	0	0
NAA10	-0.260726617	0	0
C8orf59	-0.261001662	0	0
RACK1	-0.261094323	9.02E-26	4.07E-30
PDLIM4	-0.261630466	0	0
RPS18	-0.261690589	5.96E-230	2.69E-234
ZNF511	-0.262083073	0	0
MRPS16	-0.26214194	0	0
PYURF	-0.262959256	0	0
RPL13	-0.263439026	3.06E-190	1.38E-194
EMG1	-0.263450287	0	0
AHSA1	-0.263505652	0	0
SERP1	-0.263930569	0	0
SMS	-0.264093033	0	0
TRAPPC4	-0.264340651	0	0
DBI	-0.264369028	0	0
UBE2E3	-0.264483879	0	0
HINT1	-0.265357676	3.84E-55	1.73E-59
BUD31	-0.265545108	0	0
S100A13	-0.266789184	0	0
THYN1	-0.266810519	0	0
UQCR10	-0.267126303	0	0
MRPS7	-0.267354048	0	0
HMGN3	-0.267730644	0	0
NANS	-0.268542333	0	0
NHP2	-0.268911978	0	0
ITGB3BP	-0.269140244	0	0
RUVBL1	-0.269373409	0	0
MRPL3	-0.269942898	0	0
RPL11	-0.270283284	4.74E-84	2.14E-88
OLA1	-0.270478057	0	0
TRAPPC2L	-0.270502612	0	0
TP53RK	-0.270531169	0	0
MRPL50	-0.271301291	0	0
PDLIM3	-0.271591085	0	0
TBCB	-0.27202088	0	0
PSME2	-0.272044089	0	0
RAB34	-0.272121443	0	0
VDAC1	-0.272413955	0	0
BNIP3	-0.272447766	0	0
C19orf53	-0.273354124	0	0
NT5C	-0.273717375	0	0
SSSCA1	-0.27505831	0	0

NTMT1	-0.276268973	0	0
LRR1	-0.276628114	0	0
MRPL12	-0.276845519	0	0
HNRNPF	-0.277684523	0	0
UCHL3	-0.277740184	0	0
BTF3L4	-0.278056271	0	0
RPS27A	-0.278277115	2.95E-129	1.33E-133
VDAC2	-0.278312573	0	0
RPL26	-0.278375334	1.31E-88	5.89E-93
GLRX5	-0.27855314	0	0
RPL19	-0.279518886	1.27E-118	5.74E-123
HPF1	-0.279651611	0	0
DEF8	-0.281196562	0	0
NDUFA8	-0.28129626	0	0
PRDX3	-0.281301254	0	0
PSMC4	-0.281344918	0	0
ECHS1	-0.281364672	0	0
EIF2S1	-0.28202729	0	0
CBX3	-0.28217144	0	0
CMC2	-0.282172003	0	0
TMSB4X	-0.282627806	0	0
PTS	-0.282702408	0	0
NDUFA5	-0.282941766	0	0
MYL12A	-0.28314881	9.36E-186	4.22E-190
TMEM126A	-0.283190635	0	0
MDH2	-0.283199259	0	0
EIF4A1	-0.283344266	0	0
MRPL57	-0.283478926	0	0
CDC123	-0.284155128	0	0
TIMM13	-0.284198314	0	0
CCNB2	-0.284264409	0	0
PFN1	-0.284672575	3.82E-16	1.72E-20
RPL7A	-0.284701006	1.04E-97	4.69E-102
NDUFA6	-0.285047423	0	0
NASP	-0.285733524	0	0
PCNA	-0.286268291	0	0
RPS10	-0.286298062	1	0.783829638
NDUFB3	-0.287155532	0	0
SRA1	-0.287611137	0	0
MZT2B	-0.287783986	0	0
HSPD1	-0.287936673	0	0
TIMM8B	-0.288367655	0	0
SF3B5	-0.288784673	0	0
RPL35	-0.289479253	5.85E-53	2.64E-57
ETFA	-0.29000326	0	0
CARHSP1	-0.290912868	0	0
COX7B	-0.291666632	0	0
CCT7	-0.291894598	0	0
VAPA	-0.292128773	0	0
RPS4X	-0.292170393	2.74E-128	1.24E-132
POLR2H	-0.292301838	0	0
UFD1	-0.292350392	0	0
BTF3	-0.292480472	8.13E-99	3.66E-103
CCT4	-0.292839735	0	0
PHF19	-0.29310992	0	0
GTF3C6	-0.293157126	0	0

LGALS1	-0.293275937	0	0
ADRM1	-0.293580554	0	0
EXOSC3	-0.294310257	0	0
RPS9	-0.294859047	5.39E-86	2.43E-90
CAMTA1	-0.294940089	0	0
SNU13	-0.295310012	0	0
SLIRP	-0.29554821	0	0
MRPL34	-0.295946728	0	0
DPY30	-0.29603512	0	0
POP7	-0.296063771	0	0
TK1	-0.296066285	0	0
MED10	-0.296362186	0	0
MRPS23	-0.296742406	0	0
SNRPD2	-0.296889747	0	0
PLIN3	-0.296913565	0	0
MAGOHB	-0.297263768	0	0
SEC61B	-0.297378111	0	0
RPS3	-0.297579329	9.68E-121	4.36E-125
BCAS2	-0.298054252	0	0
RPS26	-0.29908135	0	0
TRIAP1	-0.299979319	0	0
EXOSC8	-0.300359171	0	0
ARPC1A	-0.300664812	0	0
MRPS12	-0.300854275	0	0
LSM7	-0.300933611	0	0
COPE	-0.301014922	0	0
SNF8	-0.301808727	0	0
TCP1	-0.302123208	0	0
ETFB	-0.302452103	0	0
RRP36	-0.30251931	0	0
TAF9	-0.302741136	0	0
CLTA	-0.303032271	0	0
COA4	-0.30385283	0	0
VBP1	-0.303921791	0	0
H3F3A	-0.303935289	0	0
GNG5	-0.304007851	0	0
NUDCD2	-0.304226033	0	0
NUDT5	-0.304679918	0	0
MYL6	-0.304739289	2.33E-135	1.05E-139
DGUOK	-0.305019611	0	0
TXNL4A	-0.306497571	0	0
NAE1	-0.307259969	0	0
GCSH	-0.307965288	0	0
EIF6	-0.308756381	0	0
CBR1	-0.308807831	0	0
SOD1	-0.308964351	0	0
CCT3	-0.309267213	0	0
MOB4	-0.309341696	0	0
RPL6	-0.309727032	1.41E-35	6.36E-40
ANXA1	-0.309793657	0	0
SUMO2	-0.3098856	0	0
TIMM10	-0.311023614	0	0
MRPS26	-0.312233695	0	0
COX5A	-0.312647221	0	0
COX4I1	-0.313168471	0	0
GTF3A	-0.313210006	0	0

SIVA1	-0.313452198	0	0
SCAND1	-0.313782454	0	0
ECHDC1	-0.315103031	0	0
CAVIN3	-0.315292635	0	0
MRPL20	-0.315523892	0	0
HNRNPDL	-0.315551593	0	0
NDUFB4	-0.315774672	0	0
PAICS	-0.316105279	0	0
TUBB	-0.316564306	0	0
MINOS1	-0.31696888	0	0
ACP1	-0.317427974	0	0
RPL14	-0.31774637	1.69E-98	7.61E-103
RPL26L1	-0.317783092	0	0
SNRPC	-0.318019549	0	0
CHCHD1	-0.318506024	0	0
TALDO1	-0.319209663	0	0
G0S2	-0.319933207	0	0
NAA20	-0.320709361	0	0
UQCRB	-0.321119277	0	0
RPL15	-0.321334536	5.50E-179	2.48E-183
SNRNP40	-0.32150062	0	0
RPS13	-0.32166189	5.52E-177	2.49E-181
MRPL52	-0.321666323	0	0
ESD	-0.321957459	0	0
YWHAQ	-0.321998806	0	0
MRPL28	-0.322375551	0	0
RPS24	-0.324378524	5.71E-175	2.57E-179
BANF1	-0.325141663	0	0
METTL5	-0.326904873	0	0
UQCRFS1	-0.327424062	0	0
FAM96B	-0.327683623	0	0
THOC7	-0.328393618	0	0
RPL8	-0.328774721	0	0
DDX39A	-0.330956423	0	0
CYCS	-0.331585761	0	0
NOL7	-0.332657335	0	0
TWF2	-0.332870477	0	0
TXNL1	-0.333102442	0	0
POLD2	-0.333558157	0	0
RPLP0	-0.334280105	7.63E-92	3.44E-96
RPL3	-0.335725176	2.29E-154	1.03E-158
RPL5	-0.335877611	6.13E-104	2.76E-108
RPL22L1	-0.336066511	8.00E-126	3.60E-130
UQCC2	-0.336232803	0	0
MRPL27	-0.336396206	0	0
RPS3A	-0.33661645	9.66E-105	4.35E-109
FIBP	-0.336649473	0	0
TAGLN2	-0.337130407	0	0
HAT1	-0.33801778	0	0
PSMG1	-0.338089869	0	0
LSM6	-0.338270022	0	0
ARL3	-0.339037153	0	0
MRPL42	-0.339178122	0	0
SAP18	-0.340992243	0	0
LSM4	-0.341118884	0	0
HNRNPH3	-0.342405621	0	0

COPS6	-0.34258407	0	0
PDCD5	-0.342609287	0	0
SRSF2	-0.342870488	0	0
NDUFS6	-0.343304736	0	0
H2AFX	-0.343343495	0	0
HIKESHI	-0.343487852	0	0
MPG	-0.344124707	0	0
TXNDC9	-0.344147586	0	0
MRPL11	-0.344886394	0	0
PSMC6	-0.345434302	0	0
DCAF13	-0.346628598	0	0
ARPC3	-0.346985783	0	0
MAGOH	-0.347755435	0	0
UBE2I	-0.348158916	0	0
GLRX2	-0.348669835	0	0
MRPS18C	-0.348859576	0	0
MZT1	-0.348944709	0	0
PHB	-0.349641648	0	0
ZWINT	-0.349717169	1.03E-263	4.65E-268
MRPS34	-0.350337382	0	0
NDUFB8	-0.353025817	0	0
UBE2M	-0.353089466	0	0
SKP1	-0.353097392	0	0
APRT	-0.353208289	0	0
HPRT1	-0.353546507	0	0
ITGB1BP1	-0.356151311	0	0
NDUFB6	-0.356712902	0	0
ALYREF	-0.357017026	0	0
RPL27	-0.357412104	4.77E-68	2.15E-72
BAX	-0.357535723	0	0
ATP5PF	-0.358501363	0	0
MORF4L2	-0.358602126	0	0
TXN	-0.360169845	5.34E-23	2.41E-27
RPS7	-0.360505491	3.75E-260	1.69E-264
RSL24D1	-0.361343652	0	0
UBE2N	-0.361390637	0	0
KPNA2	-0.361991181	0	0
RPS19BP1	-0.364027532	0	0
NDUFS5	-0.364419211	0	0
NUDC	-0.364827047	0	0
MRPL40	-0.365346229	0	0
MRPL14	-0.366474545	0	0
UBE2T	-0.366701985	0	0
KRT10	-0.367153094	0	0
EIF3I	-0.370403724	0	0
SUMO1	-0.370751887	0	0
DYNLL1	-0.371245533	4.52E-58	2.04E-62
DNAJA1	-0.371850502	0	0
PSMC5	-0.37309633	0	0
DYNLT1	-0.373234172	0	0
HNRNPA1	-0.374179075	0	0
PSMA2	-0.375913783	0	0
VDAC3	-0.378022283	0	0
HNRNPA2B1	-0.378044918	0	0
PRMT1	-0.380233364	0	0
HMGB3	-0.380643426	0	0

NACA	-0.382279751	1.56E-62	7.01E-67
MRPL18	-0.383336102	0	0
S100A16	-0.384441482	0	0
NT5DC2	-0.384572339	0	0
VPS29	-0.384728268	0	0
UBE2V2	-0.385028273	0	0
AK6	-0.385110733	0	0
GTF2A2	-0.386726393	0	0
MRPS15	-0.387104856	0	0
CLIC1	-0.388021278	0	0
NNMT	-0.390290888	1.81E-126	8.15E-131
HMGB1	-0.390552373	0	0
S100A4	-0.390637144	0	0
PPA1	-0.390872616	0	0
SNRPF	-0.391575042	0	0
CACYBP	-0.391955807	0	0
TMEM158	-0.39245342	0	0
CCT8	-0.394850227	0	0
PSMD8	-0.394873025	0	0
H2AFV	-0.39522729	0	0
BIRC5	-0.395307107	4.11E-171	1.85E-175
ILF2	-0.396632596	0	0
EIF4EBP1	-0.396797225	0	0
PSMB5	-0.396838328	0	0
NDUFB10	-0.39880053	0	0
POMP	-0.399520628	7.95E-116	3.59E-120
RBX1	-0.399710317	0	0
TXNDC17	-0.40172639	0	0
CENPX	-0.402405018	0	0
PRDX2	-0.402795444	0	0
RPL24	-0.402823101	1.68E-282	7.56E-287
HSPB11	-0.405685774	0	0
AURKAIP1	-0.408295701	0	0
YBX1	-0.408721849	1	0.011413611
AP2S1	-0.412571467	6.49E-51	2.92E-55
LSM5	-0.413115384	0	0
LSM2	-0.414754158	0	0
GSTP1	-0.415429045	7.56E-11	3.41E-15
PSMA1	-0.415666453	0	0
MRPL47	-0.415684535	0	0
HMGA1	-0.415693636	0	0
MRPL36	-0.416096779	0	0
MALSU1	-0.416354309	0	0
DUT	-0.41641911	0	0
ENY2	-0.41712954	0	0
SNRPA1	-0.417272395	0	0
PARK7	-0.418393512	0	0
CCT5	-0.418502978	0	0
PRELID1	-0.418569645	0	0
H3F3B	-0.419724344	2.56E-248	1.15E-252
ATP5MC1	-0.420960209	0	0
NUDT1	-0.421451535	0	0
MARCKSL1	-0.422497138	0	0
DCUN1D5	-0.42256473	0	0
EBNA1BP2	-0.423702946	0	0
RPA3	-0.42378462	0	0

MRPS6	-0.42425748	0	0
SRSF9	-0.426033294	0	0
GADD45GIP1	-0.426051333	0	0
MRPL13	-0.426377117	0	0
TRMT112	-0.429524263	0	0
SNRPE	-0.431065483	0	0
NDUFAB1	-0.435067802	0	0
PSMB7	-0.435964875	0	0
PLAU	-0.436173717	0	0
RTRAF	-0.436697657	0	0
UBE2L3	-0.438827209	0	0
HMGN1	-0.439838682	0	0
PSMA4	-0.440660249	0	0
PSMA7	-0.443174417	0	0
SNRPD3	-0.445174027	0	0
NQO1	-0.445206573	0	0
EIF5A	-0.445881454	0	0
PFDN2	-0.446243303	0	0
SF3B6	-0.449167757	0	0
STRAP	-0.450532918	0	0
MRPL22	-0.450602365	0	0
GNG11	-0.450983304	0	0
CDKN3	-0.453984413	7.13E-196	3.21E-200
PSMB3	-0.457646086	0	0
SLC25A5	-0.457730185	0	0
ERH	-0.458557228	0	0
MCTS1	-0.45925153	0	0
ATP5F1C	-0.462124903	0	0
ENO1	-0.463730358	0	0
LSM3	-0.470470525	0	0
TPRKB	-0.473291082	0	0
UBB	-0.476770162	0	0
GLRX3	-0.477679445	0	0
LDHB	-0.477919343	0	0
TUBA1C	-0.478318419	0	0
PSMB6	-0.478356734	0	0
HMGB2	-0.47892742	0	0
TYMS	-0.480047521	0	0
PPP1R14B	-0.481118994	5.92E-288	2.67E-292
EEF1E1	-0.482647037	0	0
SNRPG	-0.483299314	0	0
PSMB2	-0.48695166	0	0
STOML2	-0.488834027	0	0
NPM1	-0.493593815	1.47E-48	6.64E-53
PSMA5	-0.497717912	0	0
SSBP1	-0.499408929	0	0
MRPL51	-0.504675811	0	0
DTYMK	-0.506945447	0	0
CCNB1	-0.508059636	2.29E-246	1.03E-250
TUBA1A	-0.509457104	0	0
SUB1	-0.51105555	2.85E-200	1.29E-204
PTGES3	-0.512423051	0	0
NDUFA12	-0.51275063	0	0
PPIA	-0.520846982	9.48E-77	4.27E-81
GAPDH	-0.522117594	0	0
ATP5MC3	-0.522812342	0	0

RAN	-0.523753778	1.45E-204	6.53E-209
MRPL17	-0.524685772	0	0
PSMA3	-0.5285725	0	0
SRSF7	-0.529456522	0	0
TPI1	-0.529622178	0	0
MAD2L1	-0.529707438	2.23E-295	1.01E-299
CXCL6	-0.533747995	0	0
NDUFV2	-0.534453212	0	0
PSMB1	-0.537622187	0	0
GSTO1	-0.543968486	0	0
CENPW	-0.545359995	0	0
SNRPD1	-0.552240165	0	0
SRI	-0.555806935	0	0
RBM8A	-0.556493104	0	0
CHCHD2	-0.558486524	0.019304136	8.70E-07
MRPL15	-0.571826974	0	0
SRP9	-0.573386655	0	0
C1QBP	-0.573826602	0	0
SNRPB	-0.592754614	0	0
PCLAF	-0.594880384	0	0
CKS1B	-0.597174305	0	0
PTMA	-0.602260504	3.15E-73	1.42E-77
NME1	-0.602505937	0	0
JPT1	-0.608006897	0	0
PRDX1	-0.608456022	1	0.122453743
HSPE1	-0.64874589	0	0
HMGN2	-0.673750758	0	0
RANBP1	-0.697864079	0	0
STMN1	-0.698796965	0	0
UBE2C	-0.700353845	4.63E-14	2.09E-18
FABP5	-0.718096984	0	0
SRSF3	-0.718992815	0	0
LDHA	-0.719314701	0	0
TUBA1B	-0.73609911	1.84E-38	8.30E-43
UBE2S	-0.74984822	0	0
PTTG1	-0.819426151	0	0
CKS2	-0.838048698	0	0
TUBB4B	-0.839748984	0	0
H2AFZ	-0.918940193	0.100182558	4.52E-06
